# Obese patients after gastric bypass surgery have lower brain-hedonic responses to food than after gastric banding

**DOI:** 10.1136/gutjnl-2013-305008

**Published:** 2013-08-21

**Authors:** Samantha Scholtz, Alexander D Miras, Navpreet Chhina, Christina G Prechtl, Michelle L Sleeth, Norlida M Daud, Nurhafzan A Ismail, Giuliana Durighel, Ahmed R Ahmed, Torsten Olbers, Royce P Vincent, Jamshid Alaghband-Zadeh, Mohammad A Ghatei, Adam D Waldman, Gary S Frost, Jimmy D Bell, Carel W le Roux, Anthony P Goldstone

**Affiliations:** 1 Metabolic and Molecular Imaging Group, MRC Clinical Sciences Centre, Imperial College London, Hammersmith Hospital, London, UK; 2 Section of Investigative Medicine, Division of Diabetes, Endocrinology, and Metabolism, Imperial College London, Hammersmith Hospital, London, UK; 3 Robert Steiner MRI Unit, MRC Clinical Sciences Centre, Imperial College London, Hammersmith Hospital, London, UK; 4 Department of General Surgery, Imperial Weight Centre, Imperial College Healthcare NHS Trust, London, UK; 5 Department of Gastro Surgical Research and Education, University of Gothenburg, Gothenburg, Sweden; 6 Department of Clinical Biochemistry, King's College Hospital, London, UK; 7 Division of Brain Sciences, Imperial College London, Hammersmith Hospital, London, UK; 8 Department of Experimental Pathology, UCD Conway Institute, School of Medicine and Medical Science, University College Dublin, Ireland

**Keywords:** Brain/Gut Interaction, Obesity Surgery, Brain Imaging, Bile Acid, Gastrointestinal Hormones

## Abstract

**Objectives:**

Roux-en-Y gastric bypass (RYGB) has greater efficacy for weight loss in obese patients than gastric banding (BAND) surgery. We hypothesise that this may result from different effects on food hedonics via physiological changes secondary to distinct gut anatomy manipulations.

**Design:**

We used functional MRI, eating behaviour and hormonal phenotyping to compare body mass index (BMI)-matched unoperated controls and patients after RYGB and BAND surgery for obesity.

**Results:**

Obese patients after RYGB had lower brain-hedonic responses to food than patients after BAND surgery. RYGB patients had lower activation than BAND patients in brain reward systems, particularly to high-calorie foods, including the orbitofrontal cortex, amygdala, caudate nucleus, nucleus accumbens and hippocampus. This was associated with lower palatability and appeal of high-calorie foods and healthier eating behaviour, including less fat intake, in RYGB compared with BAND patients and/or BMI-matched unoperated controls. These differences were not explicable by differences in hunger or psychological traits between the surgical groups, but anorexigenic plasma gut hormones (GLP-1 and PYY), plasma bile acids and symptoms of dumping syndrome were increased in RYGB patients.

**Conclusions:**

The identification of these differences in food hedonic responses as a result of altered gut anatomy/physiology provides a novel explanation for the more favourable long-term weight loss seen after RYGB than after BAND surgery, highlighting the importance of the gut–brain axis in the control of reward-based eating behaviour.

**Significance of this study**

**What is already known about this subject?**

- Bariatric surgery is the most effective long-term treatment for obesity.
- Gastric bypass surgery results in more weight loss than gastric banding surgery.
- Gastric bypass, but not gastric banding surgery, leads to increased postprandial anorexigenic gut hormones.
- Gastric bypass surgery patients report a shift in food preference that is away from high-calorie foods.

**What are the new findings?**

- Using functional MRI, activation in brain reward systems, including orbitofrontal cortex, amygdala, putamen, caudate and nucleus accumbens, during evaluation of the appeal of high-calorie food pictures was less after gastric bypass than after gastric banding surgery.
- High-calorie foods were less appealing and consumed less after gastric bypass than gastric banding surgery.
- These differences were not explicable by differences in hunger levels or psychological traits.
- Plasma GLP-1, PYY, bile acids and postingestive dumping symptoms were higher after gastric bypass than gastric banding surgery.

**How might it impact on clinical practice in the foreseeable future?**

- A more personalised approach to the choice of bariatric procedure including assessment of food hedonics may be warranted.
- Targeting the gut–brain food hedonic axis is important in the development of future non-surgical treatments of obesity.

## Introduction

Bariatric surgery is currently the most effective long-term treatment for obesity and its associated comorbidities.^1^ Over 20 years, Roux-en-Y gastric bypass (RYGB) surgery achieves on average 25% weight loss compared with 14% with gastric banding (BAND) surgery.^1^ This suggests that the specific anatomical manipulations of the gut in each procedure may have very different physiological effects.^2^

In RYGB, the formation of a small gastric pouch enables food to have earlier contact with the mid and distal small bowel. Food bypasses the stomach and proximal small bowel, but undiluted bile has contact with the proximal small bowel. Vagal fibres across the stomach may be disrupted.^3^ ^4^ Reduced hunger and increased satiety after RYGB are in part due to early and exaggerated responses of anorexigenic intestinal hormones, such as peptide YY (PYY) and glucagon-like polypeptide-1 (GLP-1), part of the gut–brain axis regulating ingestive behaviour.^5^ These gut hormone changes are absent after BAND surgery, where the adjustable band around the proximal stomach reduces hunger through increased intraluminal pressure on vagal afferent mechanoreceptors.^6^

Human eating behaviour is affected by hunger, and also by the reward value of food.^7^ An advantageous shift away from consumption of high-fat and sweet food after RYGB surgery has been reported in animal and human studies.^7–9^ However, differences in food hedonics between RYGB and BAND surgery, the two most commonly performed procedures around the world, and their underlying neural basis, have not been explored.

Functional MRI (fMRI) allows study of brain reward-cognitive systems related to eating behaviour by measuring regional changes in the blood oxygen level-dependent (BOLD) signal to food stimuli, a marker of neuronal activation.^10^ These include corticolimbic networks: striatal nucleus accumbens and caudate nucleus (reward conditioning, expectancy, motivation and habitual behaviour), amygdala (emotional responses to rewarding stimuli), anterior insula (integrating gustatory and other sensory information) and orbitofrontal cortex (OFC) (encoding of reward value and salience, decision making).^11^ ^12^

We hypothesised that RYGB and BAND procedures have different effects on brain reward systems, and hence, on eating behaviour, which may explain the greater weight loss seen after RYGB. We compared body mass index (BMI)-matched patients after RYGB and BAND surgery, with BMI-matched unoperated controls that had not lost weight. The primary outcome measure was reward system activation to food pictures using fMRI, and secondary outcomes were behavioural and metabolic phenotyping measures.

## Methods

Further details are given in online supplementary methods.

### Participants

Eighty-three participants (30 RYGB, 28 BAND, 25 BMI-matched (BMI-M) unoperated controls) were recruited from obesity clinics and public advertisement. Surgical patients were recruited more than 2 months after surgery, after losing at least 8% body weight (see online supplementary table S1). Unoperated BMI-matched controls were weight stable. Of these, 61 participants (21 RYGB, 20 BAND, 20 BMI-M) were eligible for a scanning visit (table 1). fMRI scans of two subjects (1 RYGB, 1 BMI-M) were excluded from analysis due to excess motion and/or poor image quality. For inclusion and exclusion criteria see online supplementary methods.

**Table 1:** Characteristics of obese patients after gastric bypass and gastric banding and unoperated controls at time of fMRI scanning

	BMI-M	BAND	RYGB	p Values*
n	20	20	21	
Age (years)	39.1±2.3 (20.0–55.0)	40.9±2.5 (22.0–59.0)	43.5±2.0 (23.0–59.0)	0.38
Gender (Male:Female)	3 : 17	1 : 19	4 : 26	0.57
Postmenopausal women, n (%)	5 (25%)	5 (25%)	6 (29%)	0.96
Ethnicity: European Caucasians, n (%)	10 (50%)	15 (75%)	16 (76%)	0.14
Preoperative BMI (kg/m^2^)	n/a	44.8 [41.9–49.2] (36.5–57.0)	48.4 [40.7–58.0] (34.7–74.6)	0.23
Current BMI (kg/m^2^)	35.4±1.9 (24.7–55.6)	35.1±1.4 (25.3–49.2)	35.3±1.7 (22.6–52.4)	0.99
Current height (m)	1.64±0.02 (1.49–1.78)	1.66±0.02 (1.53–1.79)	1.66±0.02 (1.52–1.85)	0.64
Current weight (kg)	97.0±3.1 (73.9–119.8)	97.0±3.1 (73.9–119.8)	98.1±4.9 (63.7–137.9)	0.97
Current body fat (%)	42.1±2.2 (26.0–58.2)	41.9±1.8 (23.3–54.7)	41.3±1.9 (28.4–56.0)	0.96
Weight loss (% of preoperative weight)	n/a	23.1 [14.5–29.3] (9.7–52.4)	29.9 [23.4–36.5] (16.3–40.4)	0.018 RYGB > BAND
Time since surgery (months)	n/a	9.1 [5.2–19.2] (3.6–64.6)	8.1 [5.9–11.5] (2.6–26.2)	0.25
Preoperative DM, n (%)	n/a	2 (10%)	10 (48%)	0.02 RYGB > BAND
Current DM, n (%)	2 (10%)	0 (0%)	3 (14%)	0.23
Preoperative obesity comorbidity score	n/a	6.0 [4.5–6.0] (1.0–10.0)	10.0 [6.6–11.5] (3.0–19.0)	<0.001 RYGB > BAND
Current obesity comorbidity score	0.0 [0.0–5.0] (0.0–18.0)	0.0 [0.5–2.0] (0.0–9.0)	1.0 [0.8–3.0] (0.0–10.0)	0.85
Preoperative BED, n (%)	n/a	4 (25%)	4 (19%)	1.00
Current BED, n (%)	2 (10%)	2 (10%)	1 (5%)	0.78

Data included only for those subjects who had fMRI scanning. Data presented as mean±SEM or median [IQR] for data that are not normally distributed and (range).

*p Value for overall comparison between groups.

BAND, gastric banding; BED, binge eating disorder; BMI, body mass index; BMI-M, BMI-matched; DM, type 2 diabetes mellitus; n/a, not applicable; RYGB, gastric bypass.

### Psychological and eating behaviour phenotyping

All subjects completed questionnaires to assess eating behaviour, mood, impulsivity, personality and reward sensitivity.

### fMRI protocol

**Figure 1:**
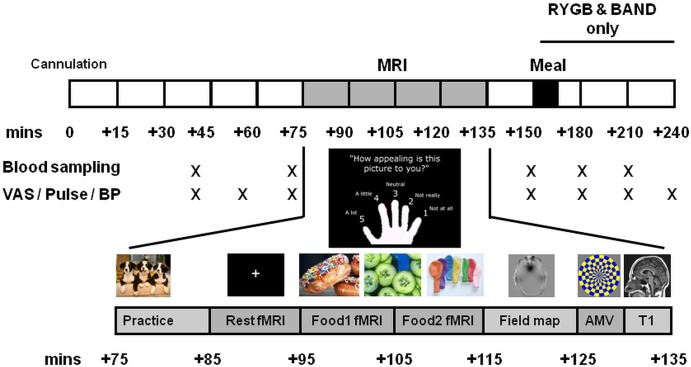
Study protocol. AMV, audio–motor–visual task; BAND, gastric banding; BP, blood pressure; fMRI, functional MRI; RYGB, gastric bypass; VAS, visual analogue scales.

Eligible subjects had structural MRI and BOLD fMRI for 1 h after an overnight fast (figure 1). During a food picture evaluation task, subjects viewed photographs of high-calorie foods, low-calorie foods, non–food-related household objects and blurred images.^13^ Subjects simultaneously rated the appeal of each picture. An auditory–motor–visual control fMRI task was performed to exclude non-specific changes in BOLD signal between groups.

### fMRI analysis

fMRI data processing used the fMRI Expert Analysis Tool V.5.98 (http://www.fmrib.ox.ac.uk/fsl). General linear model analysis was used to measure BOLD activation to (i) any food (high-calorie or low-calorie); (ii) only high-calorie foods or (iii) only low-calorie foods (compared with objects) in the food evaluation task; and for (iv) auditory, motor or visual tasks in the control paradigm.

Whole brain mixed effects analysis compared BOLD signal between surgical groups using unpaired t test, with both voxel-wise false discovery rate (FDR) corrected p<0.05 and cluster-wise threshold Z>2.1, familywise error corrected p<0.05, including age, gender and BMI as covariates. Activation in a priori functional regions of interest (fROIs) was compared between all groups for the food evaluation task using the following ROIs (see online supplementary figure S1 and table S4): bilateral OFC, amygdala, nucleus accumbens, anterior insula and caudate nucleus. These fROIs were determined from a separate cohort of 24 overweight/obese subjects who had the same fMRI protocol (see online supplementary tables S2 and S3). The anatomically constrained functional ROIs were defined by masking average activation for the food > object contrast (voxel-wise FDR, p<0.05) with the Harvard anatomical atlas (see online supplementary methods). fROIs for the control paradigm were bilateral superior posterior temporal gyrus (auditory), left precentral gyrus (motor) and bilateral lingual gyrus (visual) (see online supplementary figure S2A and table S4).

### Appetite and food palatability

Visual analogue scales (VAS) were used to measure appetite ratings, lunch palatability and other confounding symptoms (figure 1). Scanning was followed by an ad libitum ice cream test meal for the two surgical groups.

### Dietary habits

Diet macronutrient composition was assessed using 3-day self-reported home dietary records in the two surgical groups, analysed using Dietplan6 (Foresfield Software Ltd, West Sussex, UK).

### Hormonal and metabolic phenotyping

Serial blood samples before and after scanning were collected for measurement of plasma glucose, insulin, gut hormones (PYY, GLP-1 and acyl ghrelin) and bile acids (see online supplementary figure S1).

### Dumping syndrome

Symptoms and signs of dumping syndrome in the surgical groups were assessed from postprandial changes in nausea, sleepiness, blood pressure and heart rate and retrospective completion of validated questionnaires (Sigstad's and Arts’) for the 3 months following surgery.

### Statistical analysis

Results are presented as mean±SEM or median [IQR] for data that were not normally distributed. Comparisons of averages between groups used unpaired t tests or one-way analysis of variance (ANOVA) with post hoc Fisher's least significant difference test or, if not normally disturbed, Mann–Whitney U test or Kruskal–Wallis ANOVA on Ranks with post hoc Dunn's test. Comparison of prevalence between groups used χ^2^ test. Comparisons between groups for fMRI activation and eating behaviour and psychological questionnaires were adjusted for age, gender and BMI. To further investigate the link between brain responses to food cues, food hedonics and potential mediators, correlations between BOLD activation (adjusted for age, gender and BMI) and ice cream palatability or gut hormones/bile acids/dumping syndrome scores were performed to determine Pearson, or if not normally distributed Spearman, correlation coefficients. Significance was taken as p<0.05. Analyses used SPSS V.19.0 and Prism V.5.01.

## Results

### Participant characteristics

There were no significant differences between the groups in age, gender ratio, prevalence of postmenopausal women, ethnicity, current BMI, percentage body fat, prevalence of type 2 diabetes mellitus (T2DM) or binge eating disorder (BED), for both the whole cohort (see online supplementary table S1) and the scanned subjects only (table 1). The two surgical groups had similar preoperative BMI and prevalence of BED. The RYGB group had more obesity-associated comorbidities preoperatively, but not postoperatively, compared with the BAND group. There were no significant differences between the groups in any psychological questionnaire measures of depression, mood, reward sensitivity, impulsivity or personality traits (see online supplementary table S5).

### Brain activation to food pictures

**Figure 2:**
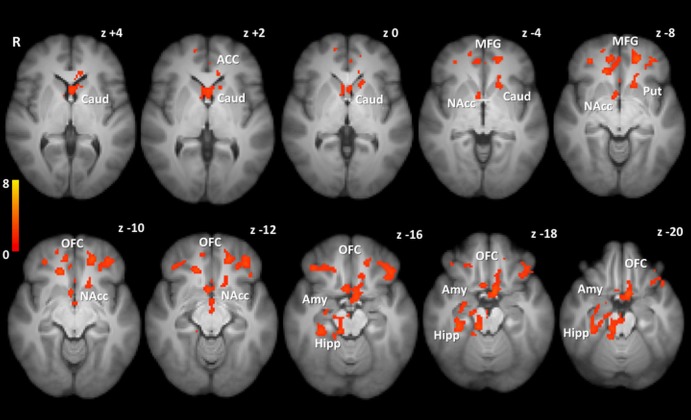
Whole brain comparison of activation to high-calorie foods between obese patients after gastric bypass and gastric banding. Whole brain group level comparison for high-calorie versus object picture contrast to demonstrate clusters in which blood oxygen level-dependent (BOLD) signal was lower in patients after gastric bypass (RYGB) compared with gastric banding (BAND) surgery, adjusting for age, gender and body mass index. No clusters showed greater activation in RYGB than BAND groups. Colour bar indicates Z values. Cluster activation thresholded at Z>2.1, familywise error p<0.05, overlaid onto the average T1 scan for all subjects (n=20 per group). Co-ordinates given in standard Montreal Neurological Institute (MNI) space. ACC: anterior cingulate cortex, Amy: amygdala, Caud: caudate, NAcc: nucleus accumbens, Hipp: hippocampus, MFG: middle frontal gyrus, OFC: orbitofrontal cortex, Put: putamen. Voxel-wise differences in BOLD activation between groups did not survive false discovery rate p<0.05 correction.

In whole brain analysis, there was lower BOLD activation in the RYGB group compared with the BAND group when viewing *high-calorie* foods in clusters within the OFC, subcallosal cortex, putamen, caudate, nucleus accumbens, hippocampus, cingulate and paracingulate gyri (figure 2, see online supplementary table S6). BOLD activation when viewing *low-calorie* foods was also lower in the OFC and subcallosal cortex in the RYGB group than in the BAND group. By contrast, there were no clusters with *greater* BOLD activation in the RYGB group compared with the BAND group when viewing high-calorie or low-calorie foods (see online supplementary table S6).

**Figure 3:**
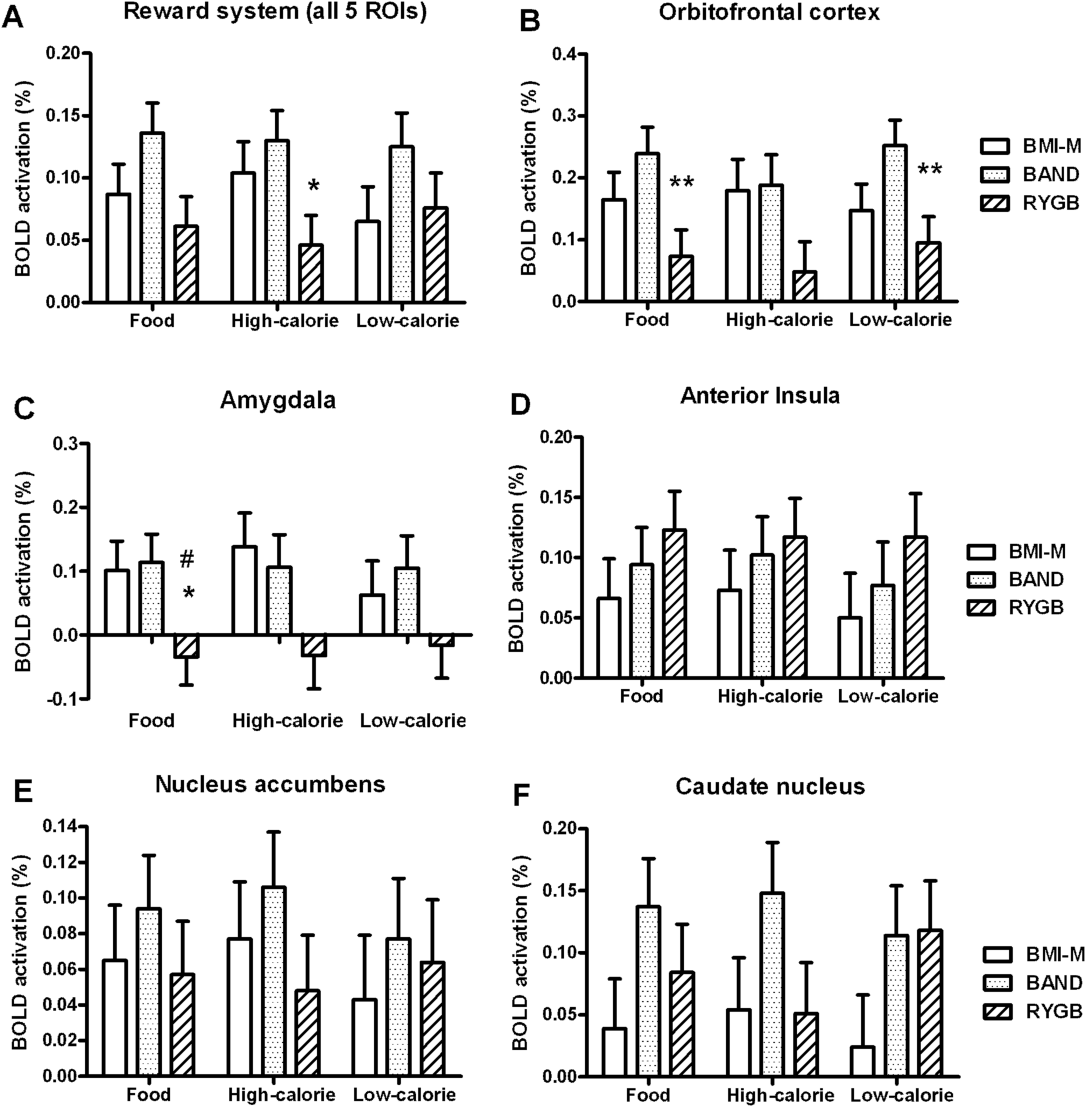
Region of interest activation to food in obese patients after gastric bypass and gastric banding and unoperated controls. Comparison of blood oxygen level-dependent (BOLD) signal to any food, only high-calorie or only low-calorie food (vs objects) in a priori functional regions of interest (fROI) between body mass index-matched unoperated controls (BMI-M, white), and obese patients after gastric banding (BAND, dotted) and gastric bypass (RYGB, striped) surgery, adjusting for age, gender and BMI. (A) Average in all five fROIs, (B) orbitofrontal cortex, (C) amygdala, (D) anterior insula, (E) nucleus accumbens, (F) caudate. Data are presented as mean±SEM. ^#^p<0.05, ^##^p<0.01, ^###^p<0.005 versus BMI-M; *p<0.05, **p<0.01, ***p<0.005 versus BAND; n=19–20 per group.

In the functional region of interest (fROI) analysis, BOLD activation within the whole reward system (average activation in the OFC, amygdala, anterior insula, nucleus accumbens and caudate) was *lower* in the RYGB group compared with the BAND group when viewing high-calorie, but not low-calorie, foods (figure 3A and see online supplementary figure S1, tables S4 and S7).

When examining individual fROIs, BOLD activation in the OFC and amygdala was lower in the RYGB group compared with the BAND group, and for amygdala also the control BMI-M group, when viewing any food (figure 3B,C and see online supplementary table S7). Similar patterns were seen for high-calorie and low-calorie foods.

There were no differences in BOLD activation of the other fROIs in the food evaluation task (figure 3D–F, see online supplementary table S7). There were also no differences in BOLD activation in the auditory, motor or visual cortices for the auditory–visual–motor control fMRI task between the three groups in either the whole brain or fROI analysis (see online supplementary figure S2A,B, tables S4 and S7).

### Food appeal scores

**Figure 4:**
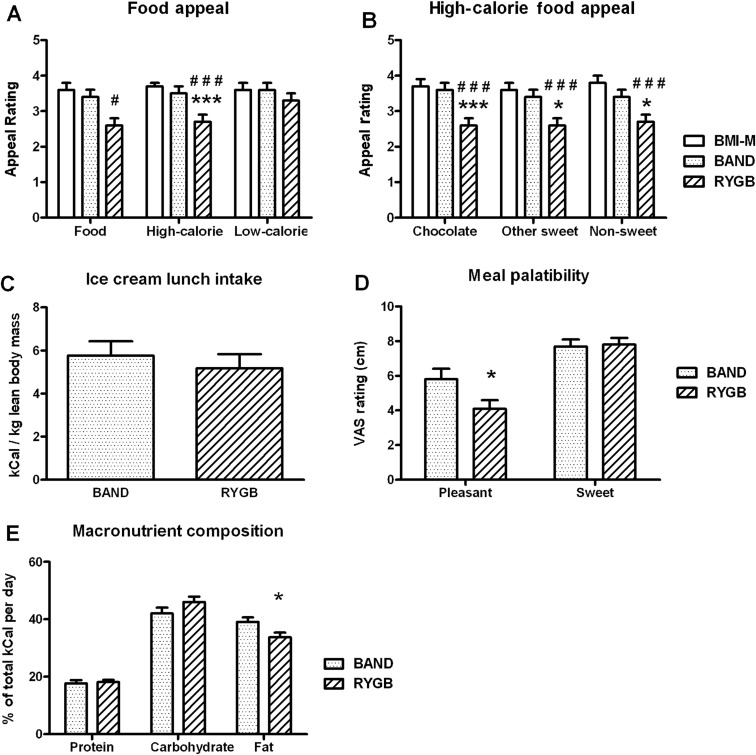
Food hedonics and dietary composition in obese patients after gastric bypass and gastric banding. Comparison of (A) appeal of any food, only high-calorie or only low-calorie food pictures; (B) appeal of subcategories of high-calorie food pictures; (C) ice cream consumption and (D) ice cream palatability rating at meal after fMRI scan; and (E) average percentage of total calories from fat from 3 day food diary, between body mass index-matched unoperated controls (BMI-M, white) and obese patients after gastric banding (BAND, dotted) and gastric bypass (RYGB, striped) surgery. Data are presented as mean±SEM. ^#^p<0.05, ^###^p<0.005 versus BMI-M; *p<0.05, ***p<0.005 versus BAND; n=20–21 per group.

During scanning, high-calorie foods, but not low-calorie foods or objects, were rated as less appealing by patients after RYGB than those after BAND surgery and control BMI-M subjects (figure 4A,B).

### Appetite VAS

**Figure 5:**
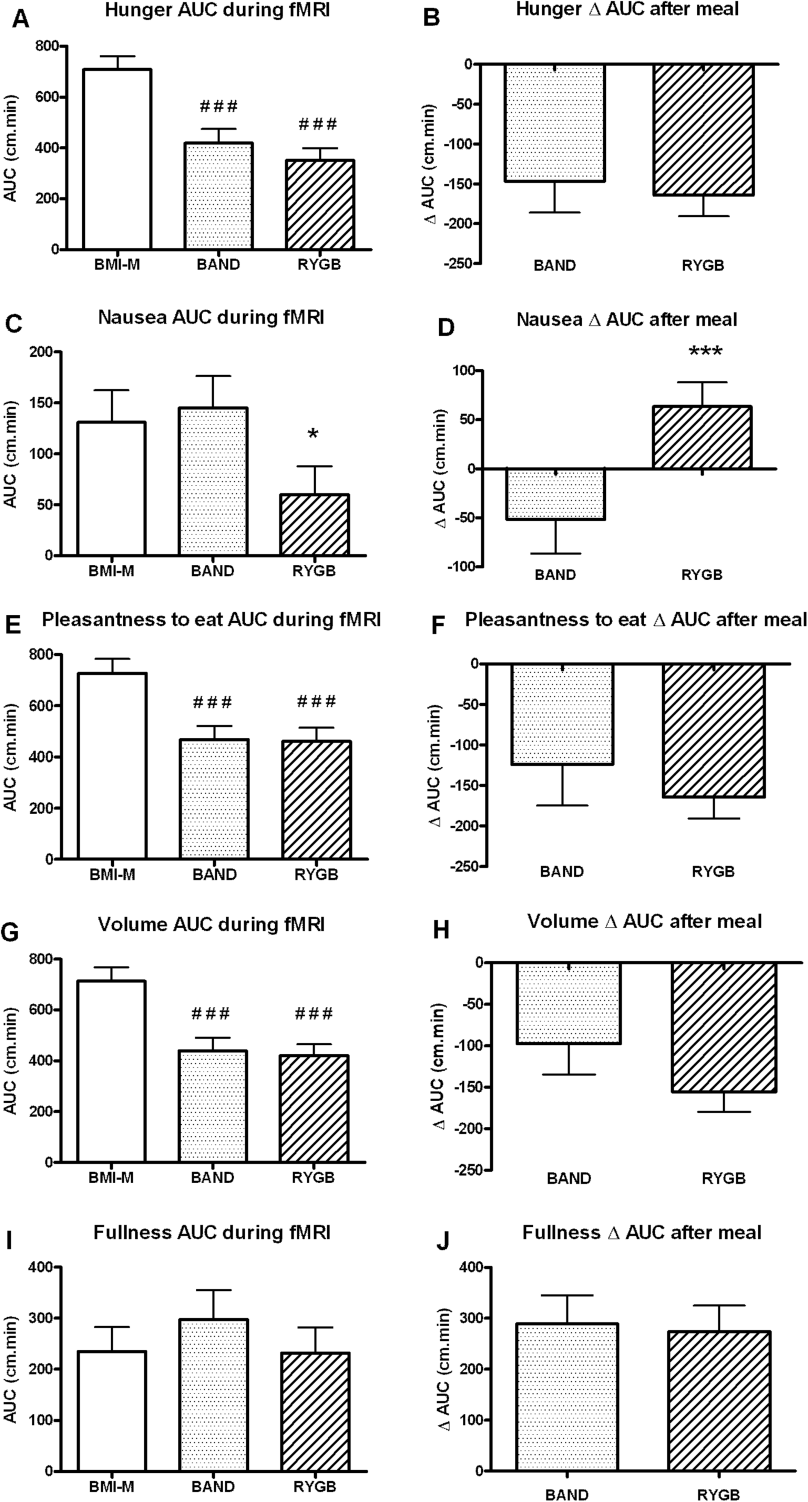
Appetite visual analogue scales during fMRI and after meal. Comparison of visual analogue scale ratings of (A and B) hunger, (C and D) nausea, (E and F) pleasantness to eat, (G and H) volume of food that could be eaten and (I and J) fullness. (A,C,E,G and I) Levels during fMRI scanning (area under curve (AUC) +40 to +150 min) between body mass index-matched unoperated controls (BMI-M, white) and obese patients after gastric banding (BAND, dotted) and gastric bypass (RYGB, striped) surgery. (B,D,F,H and J) change in levels after ice cream meal (ΔAUC +150 to +210 min) in surgical groups. Data are presented as mean±SEM. ^###^p<0.005 versus BMI-M; *p<0.05, ***p<0.005 versus BAND; n=20–21 per group.

Over the scanning period both the RYGB and BAND groups rated their ‘hunger’, ‘pleasantness to eat’ and ‘volume of food they could eat’ as lower than the control group, but there was no difference between the two surgical groups (figure 5A,E,G). RYGB patients were also less nauseated than BAND patients before the test meal, but absolute nausea ratings were still low (figure 5C).

After scanning, during a test meal, patients after RYGB and BAND surgery consumed similar amounts of ice cream (p=0.54), but patients after RYGB rated it as less ‘pleasant to eat’ than those after BAND (p=0.047), but similarly sweet (p=0.96) (figure 4C,D). The two surgical groups had similar decreases in hunger and increases in fullness after the meal (figure 5B,J).

### Dietary records

Analysis of home food diaries showed that the percentage of energy intake derived from fat was lower in patients after RYGB than after BAND surgery (figure 4E).

### Eating behaviour assessment

**Figure 6:**
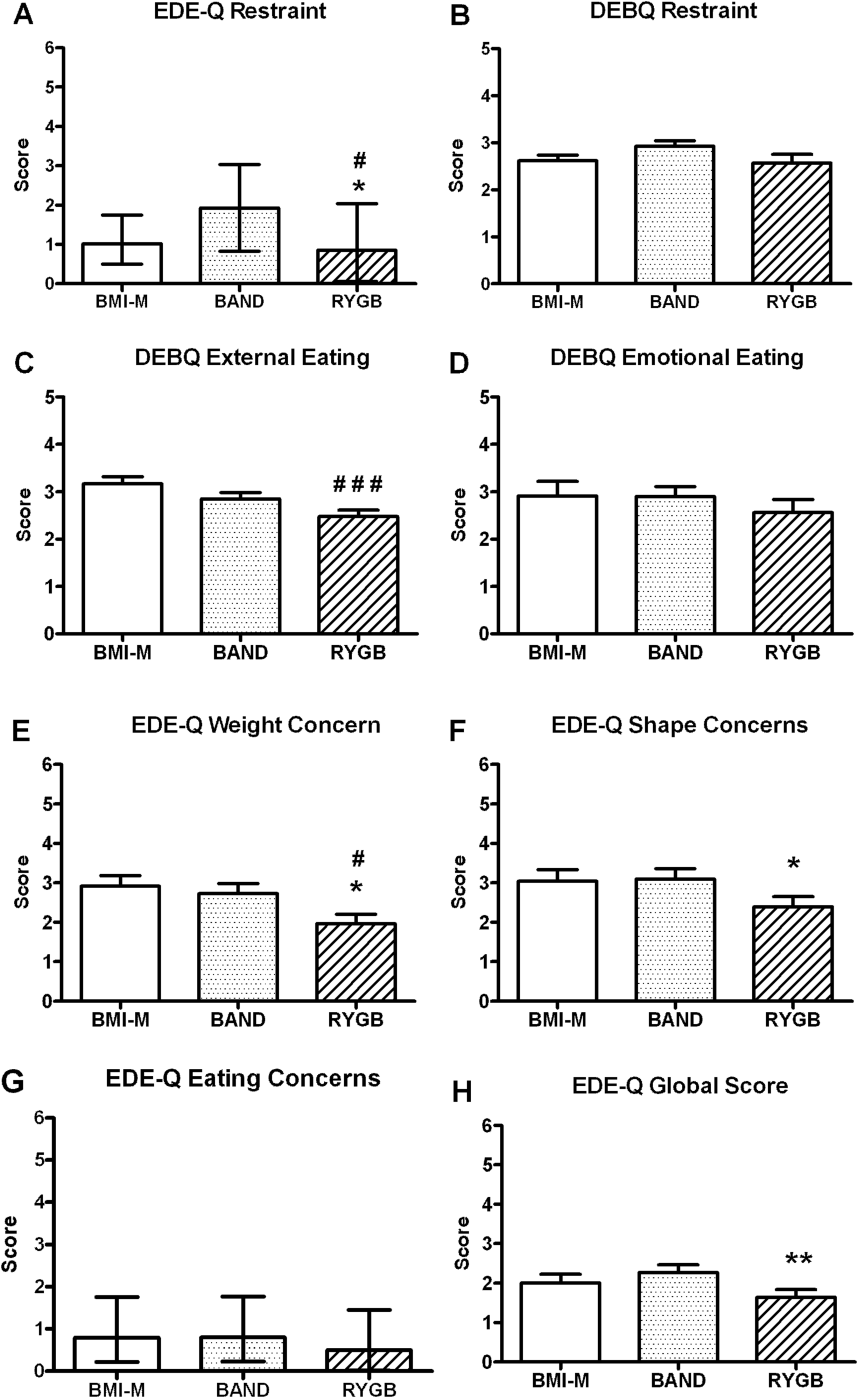
Eating behaviour. (A) EDE-Q dietary restraint, (B) DEBQ dietary restraint, (C) DEBQ external eating, (D) DEBQ emotional eating and EDE-Q (E) weight concerns, (F) shape concerns, (G) eating concerns and (H) global score of body mass index-matched unoperated controls (BMI-M, white) and obese patients after gastric banding (BAND, dotted) and gastric bypass (RYGB, striped) surgery. Data are presented as (A and G) median and IQR, or (B,C–F and H) mean±SEM. ^#^p<0.05, ^###^p<0.005 versus BMI-M; *p<0.05, **p<0.01 versus BAND; n=20–21 per group. DEBQ: Dutch Eating Behaviour Questionnaire, EDE-Q: Eating Disorders Examination Questionnaire.

In the whole cohort, eating behaviour questionnaires indicated that patients after RYGB had healthier eating behaviour and less eating disorder psychopathology compared with the BAND and/or control groups, with significantly lower scores for dietary restraint, external eating and weight and shape concerns (figure 6).

### Metabolic and hormonal phenotyping

**Figure 7:**
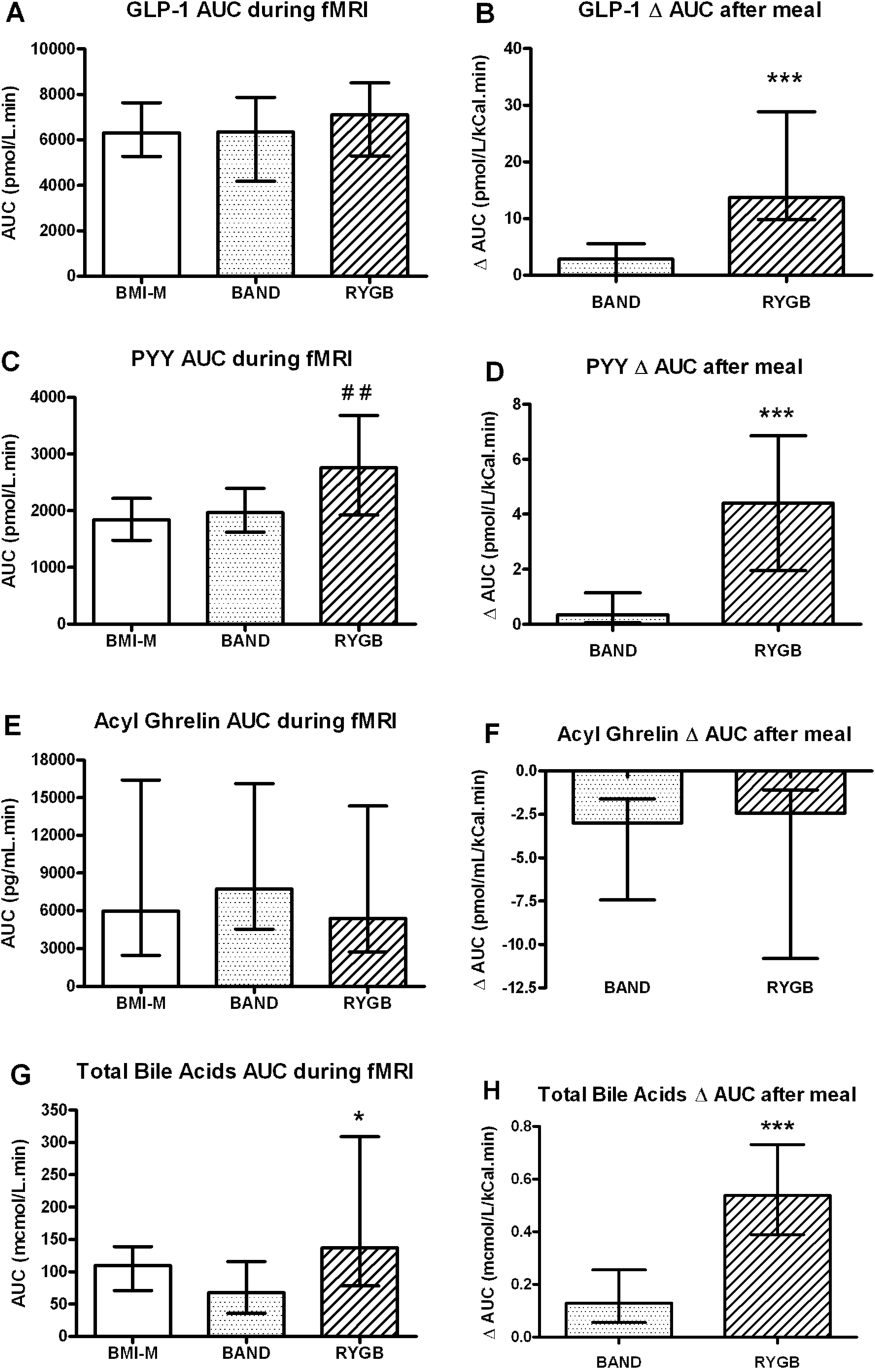
Plasma levels of gut hormones and bile acids in obese patients after gastric bypass and gastric banding and controls. Comparison of (A,C and E) plasma hormone levels (GLP-1, peptide YY, acyl ghrelin, area under curve (AUC) +40 to +150 min) and (G) total bile acid levels during fMRI scan (AUC +70 to +150 min) between body mass index-matched unoperated controls (BMI-M, white) and obese patients after gastric banding (BAND, dotted) and gastric bypass (RYGB, striped) surgery. Comparison of (B,D and F) change in plasma hormone levels and (H) change in total bile acid levels after ice cream meal (both ΔAUC +150 to +210 min) between two surgical groups. Data are presented as median and IQR. ^##^p<0.01 versus BMI-M; *p<0.05, ***p<0.005 versus BAND; n=20–21 per group.

Plasma GLP-1 levels were similar between the three groups during scanning, but increased significantly more in the RYGB group than in the BAND group after the meal (figure 7A,B). Plasma PYY levels during scanning were higher in the RYGB group than in the BMI-M group, and increased more in the RYGB group than in the BAND group after the meal (figure 7C,D). There were no differences in plasma acyl ghrelin levels between the groups (figure 7E,F).

Plasma levels of total and glycine conjugated bile acids were higher in RYGB than BAND groups both during scanning and after the meal (figure 7G,H, see online supplementary figure S3A,B). The subfractions of primary and deoxycholic bile acids were higher in the RYGB patients than the BAND patients only after the meal (see online supplementary figure S3C–F).

Plasma glucose and insulin levels during the scanning period did not differ between the two surgical groups (see online supplementary figure S3G,I). Glucose levels increased more after the meal in the RYGB group compared with the BAND group (see online supplementary figure S3H), but there were similar increases in insulin levels (see online supplementary figure S3J).

### Dumping symptoms and signs

**Figure 8:**
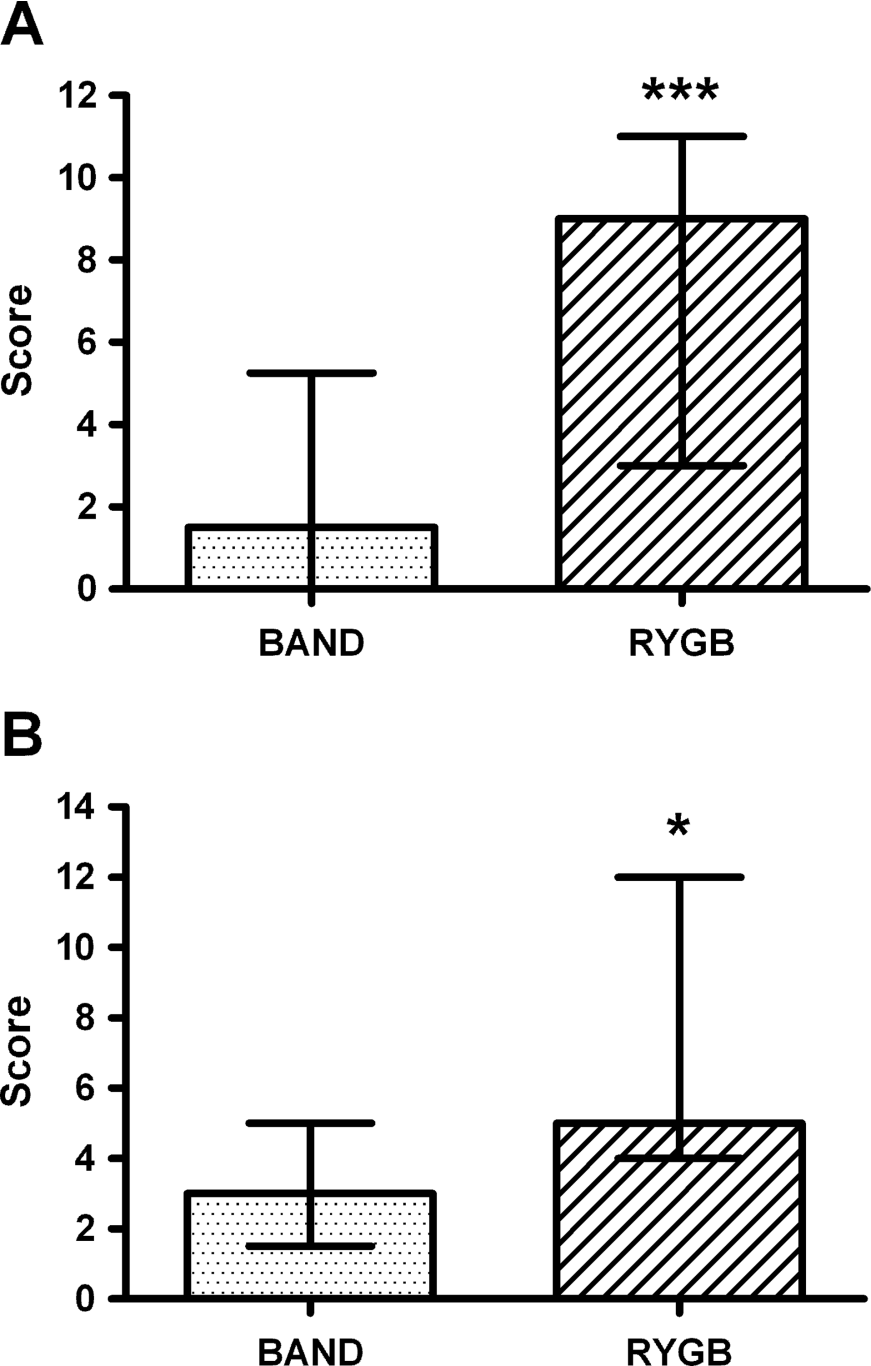
Assessment of dumping syndrome in surgical groups. Comparison of retrospective (A) Sigstad's and (B) Arts’ dumping syndrome scores during first 3 months after surgery (n=18–19 per group), between obese patients after gastric banding (BAND, dotted) and gastric bypass (RYGB, striped) surgery. Data are presented as median and IQR. *p<0.05, ***p<0.005 versus BAND.

Both retrospective dumping symptom questionnaire scores were higher for the patients after RYGB than after BAND surgery (figure 8). The RYGB group had a greater increase in symptoms of ‘feeling sick’ than the BAND group *after* the meal (figure 5D, see online supplementary table S8), but there were no differences in the change in blood pressure or heart rate after the meal between the surgical groups (see online supplementary table S8).

### Confounding variables

There were no significant differences between the groups in potential confounding factors known to affect BOLD activation to food cues or non-specifically, including sleepiness, mood, sleep duration, time since last meal or head motion during scanning (see online supplementary table S10).

### Correlation between outcome measures

BOLD activation to high-calorie food pictures in the whole reward system was positively correlated with VAS pleasantness ratings of the high-calorie ice cream lunch in the RYGB group (Pearson r=+0.49, p=0.029), and a similar trend was seen in the BAND group (r=+0.45, p=0.055).

However, within the RYGB group, there was no significant correlation between BOLD activation to any food, or high-calorie or low-calorie food pictures in the whole reward system, OFC or amygdala with any of the following secondary outcome measures: GLP-1, PYY or total bile acids area under curve (AUC) during fMRI scan (correlation coefficient −0.35 to +0.31, p=0.13–0.92); absolute GLP-1, PYY or total bile acids AUC after ice cream meal (−0.24 to +0.29, p=0.22–1.0); or either of the dumping questionnaire scores (−0.39 to +0.27, p=0.11–1.0).

## Discussion

This study has demonstrated that obese patients after RYGB have a markedly different brain-hedonic response to food compared with BAND surgery. The primary finding was that patients after RYGB had lower activation in several brain regions to food, especially high-calorie, including the OFC, amygdala, caudate nucleus, nucleus accumbens and hippocampus. These differences in brain reward systems were accompanied by beneficial differences in dietary behaviour and food hedonics, as seen from the secondary outcomes. After RYGB, patients consumed proportionately less dietary fat, found sweet, high-fat food less palatable, rated high-calorie foods as less appealing and had healthier eating behaviour than after BAND surgery. These differences were unrelated to differences in hunger or psychological traits between the surgical groups. The identification of these phenotypic differences provides a novel explanation for the more favourable long-term weight loss seen after RYGB than BAND surgery, with important clinical and pathophysiological implications.

Our finding of lower reward system activation to food pictures in the RYGB group is consistent with reduced food hedonics and consummatory behaviour. Lower neural responses to food cues in these brain regions are seen in the fed state 14–16 and are associated with decreased appeal of high-calorie food pictures,^16^ finding high-calorie foods such as ice cream less palatable (as seen in our study), sensory-specific satiety,^17^ ^18^ deliberate inhibition of the desire for pleasant foods,^19^ lower prospective food consumption,^20^ less longitudinal weight gain^21^ ^22^ and greater success in a lifestyle weight loss programme.^23^

Although some fMRI studies have shown greater activation in these regions to viewing high-calorie foods, or anticipation of food delivery, in people with obesity, higher BMI, or BED, results have been inconsistent and even contradictory.^24^ ^25^ Nevertheless, the inclusion of a lean, rather than BMI-matched control group in our study may have been helpful, to assess whether the magnitude of the reward system responses in the RYGB group are similar to those of never-obese healthy subjects.

The neuroimaging findings in this cross-sectional study in RYGB patients are consistent with prospective human studies of RYGB.^2^ A prospective fMRI study found correlations between reduced food wanting and reduced brain activation, including caudate, frontal gyri and anterior cingulate cortex, to high-calorie food in the first month after RYGB.^26^ This smaller study did not, however, control for order effects, changes in BMI or for the early postoperative dietary restrictions. By contrast, in our study, the comparison with BAND patients avoided order effects and controlled for BMI differences, and all scanning took place at least 3 months after surgery, after liquid diet restrictions had ended.

The secondary behavioural outcomes were also in agreement with prospective animal and human studies of RYGB. Animal models of RYGB show a reduced preference for sweet and/or fatty stimuli compared with sham-operated animals.^9^ ^27^ Obese patients work less hard in a progressive ratio task for sweet/fatty taste stimuli after RYGB than preoperatively.^8^ Longitudinal shifts away from a calorie-dense diet have also been described after RYGB.^28^

Metabolic phenotyping results point to potential mediators behind these differences in food hedonic responses, although direct causal inference has not been established. As expected, postprandial plasma GLP-1 and PYY gut hormone levels, and prelunch PYY levels, were higher in this cohort after RYGB than BAND and/or unoperated groups.^5^ In addition to increasing satiety through homeostatic appetite centres (vagal–brainstem–hypothalamic), these hormones also modify activity in brain reward systems and dopaminergic signalling.^29^ ^30^ GLP-1 and/or PYY acutely reduce BOLD signal to visual food cues in non-obese subjects in similar brain regions to our study,^31^ mediate changes in taste away from high-fat, sweet foods^32^ and plasma levels correlate with longitudinal reductions in uncontrolled eating after RYGB.^33^ Brain hedonic-reward systems may therefore respond not only acutely but also to chronic exposure to the repeated exaggerated postprandial increases in GLP-1 and PYY.^34^

Plasma bile acids were also higher in the RYGB than BAND group, not only postprandially but also before lunch.^35^ This could be an alternative modulator of central hedonic processing of food after RYGB. Indeed, bile acids cross the blood–brain barrier,^36^ and the bile acid receptor TGR5 is present in the brain.^37^ Bile acids also stimulate small bowel production of fibroblast growth factor 19 (FGF19), which reduces food intake centrally,^38^ and increases after RYGB.^35^ A direct role for bile acids or FGF19 after RYGB may therefore be worthy of further exploration.

RYGB patients also reported greater prevalence of symptoms consistent with dumping syndrome in the early postoperative period, and were more nauseated after ingestion of the high-calorie test meal, than after BAND surgery. Learned conditioned aversion due to postingestive effects of high-calorie foods may also therefore play a role in the reduced food hedonic responses after RYGB, potentially mediated by changes in GLP-1 and PYY.^9^

Although the orexigenic hormone ghrelin has stimulatory effects on food hedonics and reward system activation to food cues,^29^ we did not find any significant difference in plasma acyl ghrelin between surgical groups. Some studies have found reduced fasting and/or postprandial ghrelin levels after RYGB compared with before surgery or unoperated controls. This finding is, however, not universal, related to differences in surgical techniques, assay of total versus active acyl ghrelin, problems with handling and storage of plasma samples.^2^

It was not possible to further clarify which of these potential mediators might contribute to the reduced brain-hedonic response to food after RYGB, as within the RYGB group, none were correlated with BOLD activation to food cues (in those ROIs that displayed differences between surgical groups). The ability to detect such an association may have been hindered by the sample size, cross-sectional nature of the study and other physiological factors contributing to the variability in BOLD responses between individuals within the group.^39^

Although our study was cross-sectional, preoperative and postoperative confounding variables were generally similar between surgical groups, including the prevalence of BED and current T2DM. Although weight loss was greater in the RYGB patients than in the BMI-matched BAND patients, this is unlikely to explain our findings, since this would be anticipated to increase reward responses to food cues.^40^ Patient allocation to surgery was not randomised. Nevertheless, the choice of surgical procedure is not influenced by preoperative food hedonics. If anything, patients who chose RYGB tended to be heavier preoperatively and therefore less likely to have healthier food hedonics than BAND patients.

Our sample size of scanned subjects is comparable with other fMRI studies investigating food reward^10^ ^31^ and phenotyping studies after bariatric surgery,^5^ but there were a large number of outcome measures that were not corrected for multiple comparisons. We cannot, however, exclude the possibility that type 1 or 2 errors may have occurred for some results. Nevertheless, several complimentary behavioural measures showed results in the same direction as the primary fMRI endpoint.

We were surprised not to observe lower consumption of ice cream in the RYGB compared with BAND group. A possible explanation is that the test meal was not specifically designed to examine food preference, as subjects were not given a choice of foods of different caloric density. Analysis of macronutrient intake outside of the laboratory did reveal lower fat intake after RYGB compared with BAND surgery.

Our results have revealed novel differences in food reward and hedonics between these surgical treatments of obesity. This may prompt the development of more personalised approaches to surgical choices that incorporate preoperative assessment of food preference and craving. Other factors influencing the choice of bariatric procedure include local expertise and patient preference. There are potentially greater improvements in glycaemic control after RYGB,^3^ ^35^ contrasting with shorter operation time and hospital stay, lower cost and lower mortality rates with BAND surgery.^41^ However, in appropriately experienced centres, absolute mortality rates are less than 0.3% for either procedure.^41^

In conclusion, RYGB and BAND surgical treatments for obesity are distinct in their mechanisms of weight loss. Postoperatively patients have reduced hunger after both procedures, but there are lower brain hedonic and exaggerated gut hormone and bile acid responses to food after RYGB that would explain its greater efficacy for weight loss. This implicates the gut–brain axis in regulating reward-driven eating behaviour as well as homeostatic appetite, and hence, body weight. Further in-depth interrogation of these gut–brain mechanisms will accelerate development of efficacious, cheaper and safer non-surgical treatments for hedonic overeating and obesity.

## Supplementary Material

Web supplement
