# Supplementary material for: Obese patients after gastric bypass surgery have lower brain-hedonic responses to food than after gastric banding
Source: Gut. 2013 Aug 21;63(6):891–902. doi: 10.1136/gutjnl-2013-305008 (PMC4033279; doi:10.1136/gutjnl-2013-305008)
Supplement: Web supplement [file gutjnl-2013-305008-s1.pdf]

SUPPLEMENTAL INFORMATION

Obese patients after gastric bypass surgery have lower brain hedonic responses to food than after gastric banding.

Samantha Scholtz, Alexander D Miras, Navpreet Chhina, Christina G Prechtl, Michelle Sleeth, Norlida Mat Daud, Nurhafzan Ismail, Giuliana Durighel, Ahmed R Ahmed, Torsten Olbers, Royce P Vincent, Jamie Alaghband-Zadeh, Mohammad A Ghatei, Adam D Waldman, Gary S Frost, Jimmy D Bell, Carel W le Roux, Anthony P Goldstone

Supplemental Figures

Figure S1. *A priori* functional regions of interest for reward system activation during food evaluation task

Figure S2. Functional region of interest activation in auditory, motor and visual cortex during control task

Figure S3. Plasma levels of bile acid sub-fractions, glucose and insulin

Supplemental Tables

Table S1. Subject characteristics of whole cohort

Table S2. Characteristics of separate cohort of overweight/obese subjects used to create functional regions of interest in brain activation analysis

Table S3. Spatial coordinates of whole brain activation for food > objects contrast in separate cohort of overweight/obese subjects.

Table S4. Spatial coordinates of functional regions of interest in brain activation analysis

Table S5. Psychological questionnaires from subjects in whole cohort

Table S6. Spatial coordinates of whole brain comparison of activation to food between surgical groups

Table S7. Region of interest activation during food evaluation and auditory-motor-visual control task

Table S8. Assessment of dumping syndrome in surgical groups

Table S9. Potential confounding variables at scanning visit

Supplemental Methods

Participants

Inclusion and exclusion criteria

Patient characteristics

Psychological and eating behaviour questionnaires

Scanning visit protocol

fMRI protocol

fMRI confounding variables

fMRI acquisition

Food picture evaluation fMRI paradigm

Food pictures

Auditory-motor-visual control fMRI paradigm

fMRI analysis

Food palatability

Dietary habits

Metabolic, hormone and bile acid assays

Dumping symptoms

Role of funders

Supplemental References

SUPPLEMENTAL FIGURES

Figure S1. ***A priori*** **functional regions of interest for reward system activation during food evaluation task**

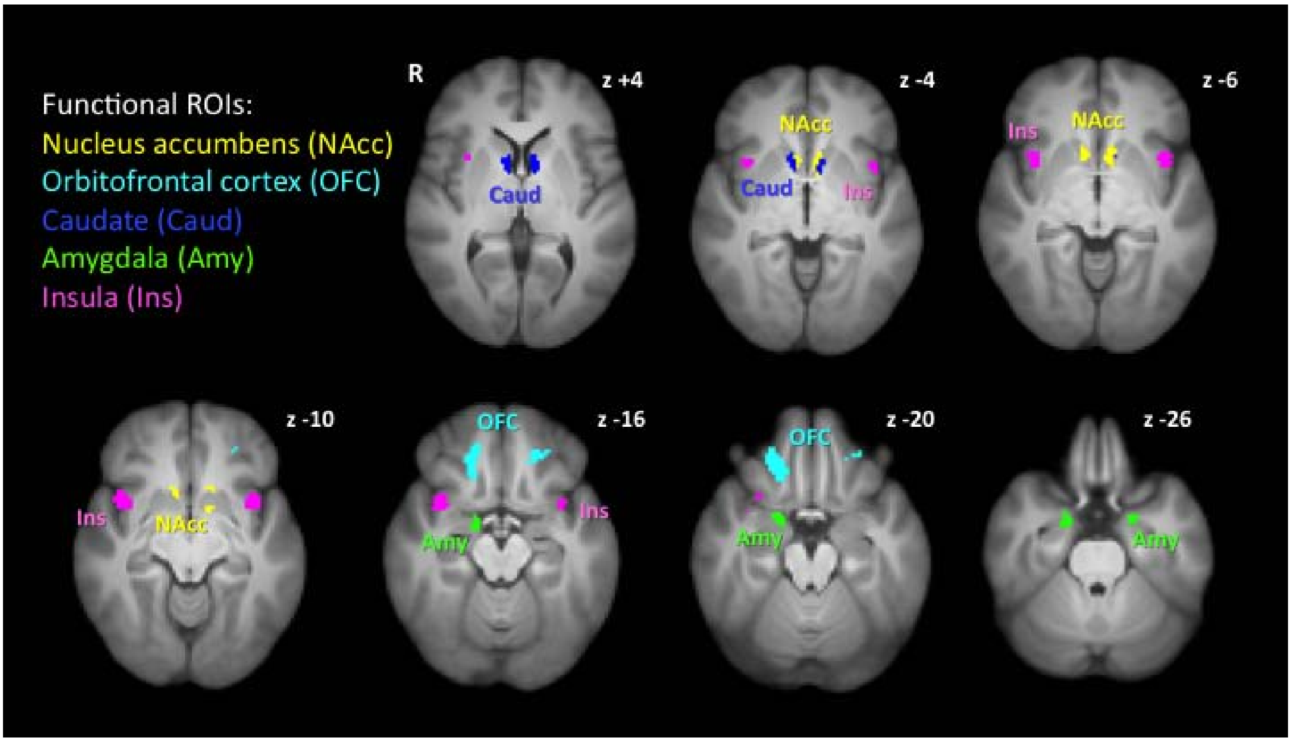

Group activation in separate cohort of obese/overweight patients for any food (high-calorie or low-calorie) vs. object picture contrast.

Activation is thresholded at voxel-wise FDR P<0.05, overlaid onto the average T1 scan for all subjects (n=24).

A priori functional regions of interest (ROIs) are indicated: nucleus accumbens (NAcc, yellow), orbitofrontal cortex (OFC, light blue), caudate (Caud, dark blue), amygdala (Amy, green), anterior insula (Ins, magenta). Co-ordinates are given in standard MNI space.

Figure S2. ***A priori*** **functional regions of interest for auditory, motor and visual cortex activation during control task**

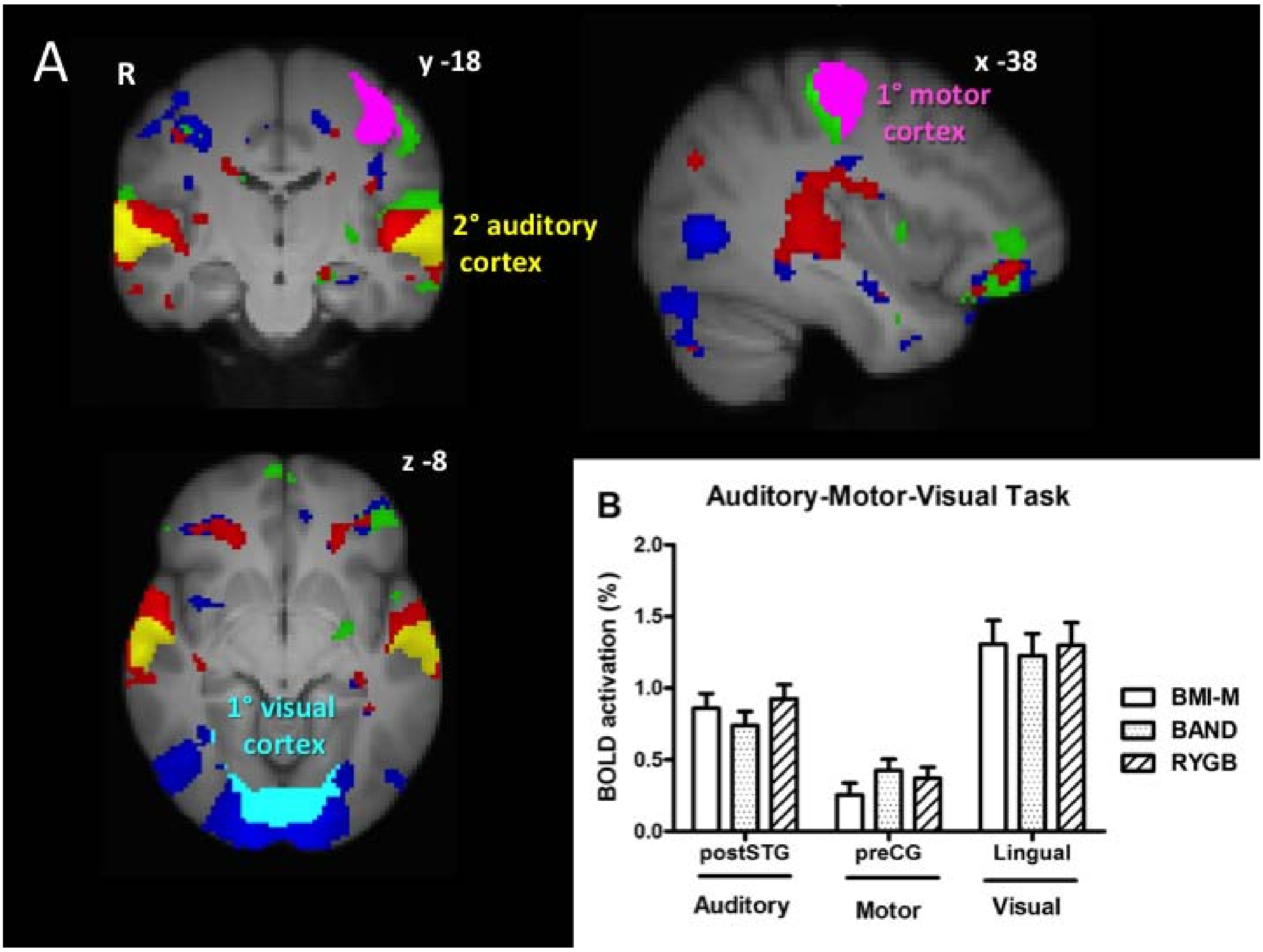

(A) Group activation maps of separate cohort of overweight/obese subjects overlaid with *a priori* anatomical regions of interest for control auditory-motor-visual task: auditory (red: listening to story) with bilateral posterior division of superior temporal gyrus (overlaid in yellow), motor task (green: button press) with left pre-central gyrus (overlaid in magenta), and visual (dark blue: flashing checkerboard) with lingual gyrus (overlaid in light blue). Activation is thresholded at voxel-wise FDR P<0.05, overlaid onto the average T1 scan for all subjects (n=24). Co-ordinates are given in standard MNI space.

(B) Comparison of BOLD signal for auditory, motor and visual control task in *a priori* functional regions of interest between body mass index-matched unoperated controls (BMI-M, white), and obese patients after gastric banding (BAND, dotted) and gastric bypass (RYGB, striped) surgery, adjusting for age, gender and BMI. Data are presented as mean ± SEM. n=19-20 per group.

Figure S3. Plasma levels of bile acid sub-fractions, glucose and insulin

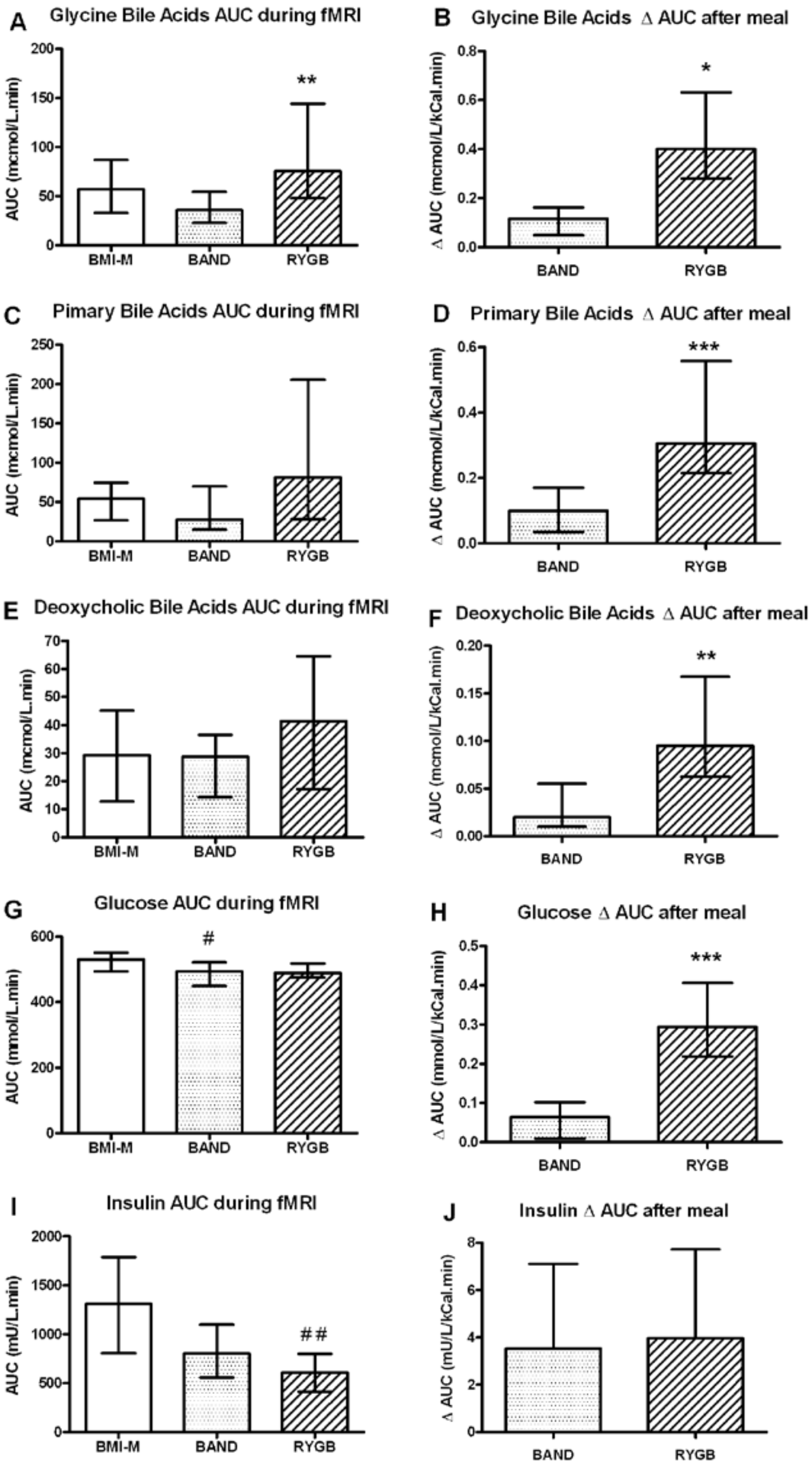

Comparison of plasma (A-F) bile acid sub-fractions (glycine, primary bile acid, deoxycholic bile acid), (G,H) glucose and (I,J) insulin levels. (A,C,E) levels during fMRI scan (area under curve (AUC) +70 to +150 mins), and (G,I) during fMRI scan (AUC +40 to +150 mins) between body mass index-matched unoperated controls (BMI-M, white), and obese patients after gastric banding (BAND, dotted) and gastric bypass (RYGB, striped) surgery. (B,D,F,H,J) change in levels after ice-cream meal (ΔAUC +150 to +210 mins) in surgical groups.

Data are presented as median and interquartile range. #P<0.05, ##P<0.01 vs. BMI-M; *P<0.05, **P<0.05, ***P<0.005 vs. BAND; n=20-21 per group.

SUPPLEMENTAL TABLES

Table S1. Subject characteristics of whole cohort

	BMI-M	BAND	RYGB	P value [a]
**n**	25	28	30	
**Age (years)**	41.0 [30.5 - 47.5]<br>(20.0 - 56.0)	42.5 [32.5 - 48.0]<br>(22.0 - 59.0)	44.5 [40.0 - 49.0]<br>(23.0 - 59.0)	0.35
**Gender (Male : Female)**	4:21	2:26	4:26	0.59
**Post-menopausal females, n (%)**	6 (24%)	6 (21%)	6 (20%)	0.95
**Ethnicity: European Caucasians, n (%)**	15 (60%)	22 (79%)	22 (73%)	0.31
**Pre-operative BMI (kg/m^2)**	n/a	46.0 [42.2 - 51.5]<br>(36.5 - 60.6)	47.6 [42.8 - 53.8]<br>(34.7 - 74.6)	0.53
**Current Weight (kg)**	99.9 [81.9 -120.9]<br>(65.5 - 168.0)	96.8 [88.3 - 106.9]<br>(68.3 - 126.3)	93.8 [84.3 - 106.2]<br>(63.6 - 144.0)	0.73
**Current BMI (kg/m^2)**	39.5 [29.3 - 44.1]<br>(24.7 - 59.5)	35.6 [32.4 - 38.2]<br>(24.8 - 50.0)	34.4 [30.2 - 38.4]<br>(23.4 - 54.2)	0.47
**Current Body fat (%)**	44.2 ± 1.9<br>(26.0 - 63.2)	43.3 ± 1.4<br>(21.7 - 54.1)	41.4 ± 2.0<br>(16.8 - 68.2)	0.54
**Weight loss (% of pre-operative weight)**	n/a	22.0 [15.2 - 29.4]<br>(8.9 - 52.4)	28.0 [23.4 - 33.0]<br>(16.3 - 40.4)	0.01<br>RYGB > BAND
**Time since surgery (months)**	n/a	15.5 [6.25 - 28.5]<br>(2 - 45)	9.75 [8 – 13]<br>(4 - 18)	0.03<br>BAND > RYGB
**Pre-operative DM, n (%)**	n/a	3 (11%)	13 (43%)	0.01<br>RYGB > BAND
**Current DM, n (%)**	3 (12%)	1 (4%)	3 (10%)	0.51
**Pre-operative obesity co-morbidity score**	n/a	6.0 [4.5 - 8.0]<br>(1.0 - 13.0)	9.0 [7.0 – 11.0]<br>(2.0 - 19.0)	0.001<br>RYGB > BAND
**Current obesity co-morbidity score**	2.0 (0.0 - 7.3)<br>(0.0 - 18.0)	1.5 [1.0 - 2.5]<br>(0.0 - 9.0)	1.0 [0.0 - 2.0]<br>(0.0 - 10.0)	0.60
**Pre-operative BED, n (%)**	n/a	7 (25%)	9 (30%)	0.90
**Current BED, n (%)**	4 (16%)	2 (7%)	1 (3%)	0.23

Data included for the whole cohort. Data presented as mean ± SEM or median [interquartile range] for data that is not normally distributed, and (range).

[a] P value for overall comparison of averages or prevalence between groups.

Abbreviations: BAND: gastric banding, BED: binge eating disorder, BMI: body mass index, BMI-M: BMI-matched, DM: type 2 diabetes mellitus, n/a: not applicable, RYGB: gastric bypass.

Table S2. Characteristics of separate cohort of overweight/obese subjects used to create functional regions of interest in brain activation analysis.

**n**	24
**Age (years)**	29.0 [26.0 - 38.5]<br>(20.0 - 48.0)
**Gender (Male : Female)**	6:18
**Ethnicity: European Caucasians, n (%)**	14 (58%)
**Current BMI (kg/m^2)**	30.7 [26.3 - 32.8]<br>(25.4 - 42.7)
**Current body fat (%)**	36.3 ± 2.0<br>(17.1 - 54.5)
**Current DM, n (%)**	0 (0%)
**Current obesity co-morbidity score**	0.0 [0.0 - 0.0]<br>(0.0 - 8.0)
**Duration fasting (hours)**	15.9 [1516.8]<br>(13.7 - 19.7)

Data presented as mean ± SEM or median [interquartile range] for data that is not normally distributed, and (range).
Abbreviations: BMI: body mass index, DM: type 2 diabetes mellitus.

Table S3. Spatial co-ordinates of whole brain activation for food > objects contrast in separate cohort of overweight/obese subjects.

Contrast	Number of voxels	Z statistic	x	y	z	Brain region
**Any food (high-calorie or low-calorie)**	11961	6.85	8	-84	-6	R lingual gyrus
**> object**	2416	5.08	40	8	-14	L insula cortex / temporal pole
	504	4.29	4	26	26	R cingulate gyrus
	358	4.32	-22	-56	40	L superior parietal lobe/ lateral occipital cortex
	322	4.8	-36	-8	8	L insula cortex
	199	3.88	40	38	8	R frontal pole/ inferior frontal gyrus
	187	4.01	-20	-26	-12	L hippocampus/ parahippocampal gyrus
	184	3.84	48	10	20	R inferior frontal gyrus/ precentral gyrus
	149	3.66	4	-30	26	R cingulate gyrus
	131	3.83	-6	2	28	L cingulate gyrus
	105	3.34	52	-24	44	R postcentral gyrus
	102	3.42	28	-4	46	R precentral gyrus / middle frontal gyrus
	101	3.51	-14	-68	-48	L cerebellum
	93	4.05	-48	-18	42	L postcentral gyrus / precentral gyrus
	84	4.28	-52	-44	-22	L inferior temporal gyrus
	63	3.6	-20	38	-14	L frontal pole / orbitofrontal cortex
	51	3.56	-18	-44	-44	L cerebellum
	44	3.41	24	-34	-50	L cerebellum
	43	3.48	22	-68	-54	L cerebellum
	36	3.41	-18	-14	4	L thalamus
	35	3.34	-26	-46	-56	L cerebellum
	29	3.07	-40	-40	40	L supramarginal gyrus / superior parietal lobe
	28	3.43	14	38	36	R frontal pole
	26	3.31	-66	-14	2	L superior temporal gyrus
	24	3.1	12	4	32	R cingulate gyrus

	24	3.21	-60	-24	24	L supramarginal gyrus / postcentral gyrus
	23	3.18	8	-58	66	R precuneus / superior parietal lobe / lateral occipital
	22	3.11	8	64	2	R frontal pole
	19	3.23	-2	-24	-38	L brainstem
	19	3.03	-42	-58	-46	L cerebellum
	17	3.15	52	-20	24	R parietal operculum / supramarginal gyrus /
	15	3.33	60	-50	-22	R inferior temporal gyrus

Stereotactic coordinates (x, y, z given in standard MNI space) for peak voxel within each cluster at group level activation, adjusting for age, gender and BMI, thresholded at voxel-wise FDR P<0.05 (n=24), and cluster size > 10 voxels.

Table S4. Spatial coordinates of functional regions of interest in brain activation analysis.

Functional region of interest	Hemisphere	Number of voxels	Z statistic	x	y	z
**Food vs. Object contrast**						
Orbitofrontal cortex	Right	170	3.81	18	36	-18
	Left	63	3.60	-20	38	-14
Amygdala	Right	110	3.85	18	0	-26
	Left	16	3.99	-18	0	-26
Nucleus Accumbens	Right	62	3.45	8	14	-4
	Left	91	4.11	-6	10	-2
Anterior Insula	Right	188	5.08	40	8	-14
	Left	116	4.43	-38	8	-12
Caudate	Right	129	3.88	8	6	2
	Left	74	4.18	-6	-6	0
**Auditory task**						
Posterior division of superior temporal gyrus	Right	1109	5.56	64	-14	4
	Left	1108	5.39	-62	-22	2
**Motor task**						
Precentral gyrus	Left	873	5.78	-36	-24	56
**Visual task**						
Lingual gyrus	Bilateral	1412	5.59	6	-90	-10

Stereotactic coordinates (x, y, z given in standard MNI space) for peak voxel of group activation, adjusting for age, gender and BMI, thresholded at voxel-wise FDR $P<0.05$ (n=24).

Table S5. Psychological questionnaires from subjects in whole cohort

	BMI-M	BAND	RYGB	P value [a]
n	25	28	30	
**Beck Depression Inventory II** (score/63)	8.0 [2.0 - 14.0] (1.0 - 44.0)	6.0 [3.0 -14.5] (1.0 - 38.0)	4.5 [2.0 - 11.0] (0.0 - 32.0)	0.99
Moderate-severe depression (>15), n (%)	5 (20%)	7 (25%)	7 (23%)	0.22
**On antidepressants treatment, n (%)**	3 (12%)	5 (18%)	8 (27%)	0.38
**PANAS**				
Negative affect (score /50)	18.0 [12.5 - 24.3] (10.0 - 43.0)	15.0 [13.0 - 20.5] (9.0 - 33.0)	15.0 [12.0 - 18.0] (10.0 - 35.0)	0.67
Positive affect (score /50)	32.3 ± 1.7 (18.0 - 49.0)	30.6 ± 2.0 (15.0 - 49.0)	32.8 ± 1.7 (12.0 - 47.0)	0.63
**Behavioural activation and inhibition scale**				
BAS drive (score /16)	11.0 [9.0 - 13.0] (7.0 -15.0)	10.0 [8.5 - 11.5] (5.0 - 15.0)	10.0 [7.0 - 12.0] (4.0 - 16.0)	0.35
BAS reward responsiveness (score /20)	18.0 [15.8 - 19.0] (9.0 -20.0)	17.0 [15.0 - 19.5] (8.0 - 20.0)	17.0 [14.0 - 19.0] (11.0 - 20.0)	1.00
BAS fun-seeking (score /16)	12.1 ± 0.4 (8.0 - 16.0)	11.6 ± 0.4 (7.0 - 16.0)	11.0 ± 0.5 (5.0 - 16.0)	0.32
BIS (score /28)	21 [17.8 -24.0] (11.0 - 28.0)	21.5 [19.0 - 22.5] (11.0 - 28.0)	20.0 [18.0 - 21.0] (12.0 -28.0)	0.87
**Impulsivity**				
Barratt impulsivity scale (score /120)	60.5 ± 2.4 (30.0 -77.0)	66.6 ± 2.6 (45.0 - 99.0)	63.2 ± 2.4 (25.0 - 93.0)	0.20
**EPQ-R**				
Extraversion (score /23)	14.9 ± 0.9 (2.0 - 22.0)	14.2 ± 1.0 (5.0 - 23.0)	13.7 ± 1.0 (4.0 - 23.0)	0.49
Psychoticism (score /32)	6.4 ± 0.6 (0.0 - 13.0)	6.6 ± 0.5 (2.0 - 13.0)	5.4 ± 0.6 (1..0 - 13.0)	0.35
Neuroticism (score /24)	12.9 ± 0.9 (6.0 - 23.0)	11.9 ± 1.3 (1.0 - 24.0)	12.6 ± 1.0 (2.0 - 24.0)	0.73
Lying (score /21)	8.7 ± 1.0(1.0 - 17.0)	9.6 ± 0.7 (3.0 - 17.0)	9.8 ± 0.8 (0.0 - 18.0)	0.83

Data included for the whole cohort. Data presented as mean ± SEM or median [interquartile range] for data that is not normally distributed, and (range), adjusted for age gender and BMI.

[a] P value for overall comparison of averages or prevalence between groups.

Note that similar results were obtained when limiting the analysis to the scanned subjects only (data not shown).

Abbreviations: BAND: gastric banding, BAS/BIS: Behavioural Activation and Inhibition Scale, BMI-M: body mass index matched, EPQ-R: Eysenck Personality Questionnaire, PANAS: Positive and Negative Affect Schedule, RYGB: gastric bypass.

Table S6. Spatial coordinates of whole brain comparison of activation to food between surgical groups.

Contrast	Number of voxels	Z statistic	x	y	z	Brain region
**GASTRIC BANDING > GASTRIC BYPASS**						
**Any food (high-calorie or low-calorie) > object**	Cluster 1 - 1470	4.12	16	30	-12	Right orbitofrontal cortex
		3.69	-18	44	-8	Left orbitofrontal cortex
		3.61	-6	8	-20	Left orbitofrontal cortex
		3.45	-16	40	-14	Left orbitofrontal cortex
		3.42	16	16	-18	Right orbitofrontal cortex
		3.20	0	22	-8	Right orbitofrontal cortex
		3.18	4	10	-14	Right subcallosal cortex
		2.93	38	34	-16	Right orbitofrontal cortex / subcallosal cortex
		2.89	-8	18	-20	Left orbitofrontal cortex / subcallosal cortex
		2.83	-16	18	-8	Left putamen / caudate / nucleus accumbens
**High-calorie food > object**	Cluster 1 - 980	4.05	-38	18	-30	Left temporal cortex
		3.55	-18	44	-10	Left orbitofrontal cortex
		3.51	16	30	-10	Right orbitofrontal cortex
		3.21	-42	26	-14	Left orbitofrontal cortex
		3.17	40	34	-14	Right orbitofrontal cortex
		3.12	-36	38	-12	Right orbitofrontal cortex
		3.04	32	42	-8	Right orbitofrontal cortex / frontal pole
		3.03	-42	30	-16	Left orbitofrontal cortex / frontal pole
		3.00	10	46	-8	Right cingulate/paracingulate gyrus
		2.92	-34	44	-8	Left frontal pole
	Cluster 2 - 1232	3.54	-6	6	-18	Left subcallosal cortex
		3.28	10	-32	-18	Right brainstem
		3.22	4	10	-14	Right subcallosal cortex
		3.21	32	-32	-18	Right hippocampus
		3.05	10	-22	-24	Right brainstem
		3.04	2	-22	-22	Right brainstem
		2.89	-16	18	-8	Left putamen / caudate / nucleus accumbens
		2.88	12	-40	-22	Left brainstem

Contrast	Number of voxels	Z statistic	x	y	z	Brain region
**Low-calorie food > object**	Cluster 1 - 1041	3.95	14	30	-12	Right orbitofrontal cortex
		3.46	-16	40	-14	Left orbitofrontal cortex
		3.43	4	22	-8	Right subcallosal cortex
		3.32	-4	8	-18	Left subcallosal cortex
		3.25	16	16	-18	Left orbitofrontal cortex
		3.20	-16	46	-6	Left orbitofrontal cortex
		3.17	12	8	-18	Right orbitofrontal cortex / subcallosal cortex
		3.02	-6	18	-18	Left subcallosal cortex
		3.01	-18	42	-20	Left orbitofrontal cortex / frontal pole
		2.94	-8	12	-22	Left orbitofrontal cortex / subcallosal cortex
**GASTRIC BYPASS > GASTRIC BANDING**						
**Any food (high-calorie or low-calorie) > object**						Nil significant
**High-calorie food > object**						Nil significant
**Low-calorie food > object**						Nil significant

Stereotactic coordinates (x, y, z) for peak voxel of group activation for food category vs. objects, adjusted for age, gender and BMI, cluster thresholded at Z>2.1, FWE P<0.05 (n=20 per group), given in standard MNI space. Voxel-wise differences in BOLD activation between groups did not survive FDR P<0.05 correction.

Table S7. Region of interest activation during food evaluation and auditory-motor-visual control task.

Region of interest	Contrast [b]	BMI–M	BAND	RYGB	P value [a]
n		19	20	20	
FOOD EVALUATION TASK					
Reward system (all 5 ROIs)	Food	0.082 ± 0.029<br>(-0.127 to 0.335)	0.138 ± 0.020<br>(0.005 to 0.340)	0.064 ± 0.021<br>(-0.101 to 0.225)	0.08<br>BAND > RYGB 0.03
	High-calorie	0.100 ± 0.027<br>(-0.152 to 0.294)	0.131 ± 0.022<br>(-0.012 to 0.372)	0.049 ± 0.023<br>(-0.176 to 0.235)	0.05<br>BAND > RYGB 0.02
	Low-calorie	0.060 ± 0.033<br>(-0.150 to 0.348)	0.128 ± 0.026<br>(-0.042 to 0.472)	0.078 ± 0.022<br>(-0.060 to 0.253)	0.28
Orbitofrontal cortex	Food	0.177 ± 0.050<br>(-0.064 to 0.878)	0.235 ± 0.040<br>(-0.121 to 0.543)	0.066 ± 0.040<br>(-0.459 to 0.306)	0.029<br>BAND > RYGB 0.008
	High-calorie	0.191 ± 0.060<br>(-0.099 to 0.853)	0.182 ± 0.044<br>(-0.285 to 0.474)	0.043 ± 0.045<br>(-0.357 to 0.478)	0.09
	Low-calorie	0.160 ± 0.046<br>(-0.076 to 0.793)	0.250 ± 0.038<br>(-0.04 to 0.646)	0.085 ± 0.042<br>(-0.498 to 0.372)	0.03<br>BAND > RYGB 0.01
Amygdala	Food	0.086 ± 0.051<br>(-0.172 to 0.592)	0.121± 0.035<br>(-0.187 to 0.543)	-0.027 ± 0.047<br>(-0.694 to 0.243)	0.04<br>BAND > RYGB 0.02
	High-calorie	0.124 ± 0.056<br>(-0.187 to 0.787)	0.110 ± 0.046<br>(-0.345 to 0.527)	-0.023 ± 0.055<br>(-0.690 to 0.298)	0.059
	Low-calorie	0.049 ± 0.056<br>(-0.263 to 0.624)	0.114 ± 0.039<br>(-0.087 to 0.589)	-0.011 ± 0.056<br>(-0.633 to 0.425)	0.24
Nucleus accumbens	Food	0.061 ± 0.035<br>(-0.21 to 0.356)	0.097 ± 0.024<br>(-0.058 to 0.259)	0.060 ± 0.030<br>(-0.182 t0 0..333)	0.67
	High-calorie	0.075 ± 0.034<br>(-0.295 to 0.376)	0.107 ± 0.026<br>(-0.063 to 0.367)	0.048 ± 0.032<br>(-0.281 to 0.297)	0.43
	Low-calorie	(0.038 ± 0.038)<br>(-0.28 to 0.298)	0.080 ± 0.033<br>(-0.209 to 0.428)	0.065 ± 0.031<br>(-0.217 to 0.454)	0.79

Region of interest	Contrast [b]	BMI–M	BAND	RYGB	P value [a]
**Anterior Insula**	**Food**	0.0534 ± 0.025<br>(-0.212 to 0.256)	0.095 ± 0.034<br>(-0.094 to 0.496)	0.134 ± 0.037<br>(-0.218 to 0.532)	0.47
	**High-calorie**	0.062 ± 0.032<br>(-0.237 to 0.254)	0.102 ± 0.028<br>(-0.132 to 0.336)	0.127 ± 0.037<br>(-0.240 to 0.468)	0.64
	**Low-calorie**	0.038 [-0.058 to 0.107]<br>(-0.148 to 0.310)	0.051 [-0.034 to 0.106]<br>(-0.181 to 0.678)	0.129 [0.040 to 0.182]<br>(-0.192 to 0.545)	0.43
**Caudate**	**Food**	0.031 ± 0.051<br>(-0.371 to 0.638)	0.141 ± 0.033<br>(-0.059 to 0.605)	0.087 ± 0.032<br>(-0.100 to 0.411)	0.23
	**High-calorie**	0.040 [-0.045 to 0.177]<br>(-0.403 to 0.595)	0.013 [0.081 to 0.197]<br>(-0.094 to 0.733)	0.038 [-0.057 to 0.150]<br>(-0.189 to 0.415)	0.15
	**Low-calorie**	0.025 [-0.120 to 0.117]<br>(-0.375 to 0.639)	0.075 [0.019 to 0.166]<br>(-0.117 to 0.488)	0.010 [0.017 to 0.170]<br>(-0.075 to 0.432)	0.15
**CONTROL AMV TASK**					
**Combined (all 3 ROIs)**		0.816 ± 0.089<br>(0.221 - 1.815)	0.856 ± 0.077<br>(0.323 - 1.605)	0.798 ± 0.068<br>(0.415 - 1.331)	0.85
**Posterior division superior temporal gyrus**	**Auditory**	0.853 ± 0.134<br>(0.168 to 2.172)	0.942 ± 0.117<br>(0.065 to 2.098)	0.728 ± 0.074<br>(0.288 to 1.443)	0.41
**Left precentral gyrus**	**Motor**	0.276 ± 0.104<br>(-0.807 to 0.846)	0.415 ± 0.077<br>(-0.076 to 0.973)	0.360 ± 0.057<br>(-0.049 to 0.727)	0.33
**Lingual gyrus**	**Visual**	1.320 ± 0.169<br>(0.156 to 2.906)	1.212 ± 0.152<br>(0.152 to 2.739)	1.304 ± 0.146<br>(0.357 to 2.581)	0.92

Average group activation in separate and combined *a priori* regions of interest (ROI) for food category vs. objects during food evaluation task, or auditory, motor or visual cortex during control task, adjusted for age, gender and BMI. Data presented as mean ± SEM and (range).

[a] P value for overall comparison of averages between groups using ANOVA, with post-hoc comparison given beneath.

[b] Contrasts with food pictures are compared to object pictures.

Abbreviations: AMV: auditory-motor-visual, BAND: gastric banding, BMI-M: body mass index matched, RYGB: gastric bypass.

Table S8. Assessment of dumping syndrome in surgical groups.

	BAND	RYGB	P value
**n**	20	21	
**Sigstad's score [a]**	1.5 [0.0 - 5.0]<br>(-4.0 to 11.0)	9.0 [3.0 - 11.0]<br>(0.0 -29.0)	0.002<br>RYGB > BAND
**Arts' score [a]**	3.0 [2.0 - 5.0]<br>(0.0 - 8.0)	5.0 [4.0 - 12.0]<br>(0.0 - 24.0)	0.02<br>RYGB > BAND
**Δ Heart rate (beats per minute)**	7.9 ± 1.4<br>(-6.0 to 20.0)	5.3 ± 1.7<br>(-7.0 to 21.0)	0.24
**Δ Systolic BP (mm Hg)**	-2.4 ± 3.8<br>(-23.0 to 38.0)	-10.7 ± 3.4<br>(-40.0 to 19.0)	0.11
**Δ Diastolic BP (mm Hg)**	-2.5 ± 2.9<br>(-28.0 to 17.0)	-3.7 ± 1.8<br>(-16.0 to 10.0)	0.72
**VAS Sleepiness**			
After meal Δ AUC (cm.min)	0.0 [-78.0 to 28.5]<br>(-396.0 to 442.5)	-30.0 [-113.6 to 3.0]<br>(-217.5 to 63.0)	0.34
**VAS Nausea**			
After meal Δ AUC (cm.min)	-19.5 [-69.8 to 0.0]<br>(-549.0 to 186.0)	9.0 [0.0 to 79.1]<br>(-10.5 to 408.0)	<0.001<br>RYGB > BAND

Data presented as mean ± SEM or median [interquartile range] for data that is not normally distributed, and (range).

[a] n=18-19 per group

Δ heart rate and blood pressure: change between time points +150 and +210 min. Δ AUC for VAS: change in AUC between time points +150 to +210 min.

Abbreviations: AUC: area under the curve BAND: gastric banding group, BMI-M: body mass index matched group, BP: blood pressure, mm: millimeters, RYGB: gastric bypass, VAS: visual analogue scale.

Table S9. Potential confounding variables at scanning visit.

	BMI-M	BAND	RYGB	P value [a]
**n**	20	20	21	
**PANAS positive (score /50)**	32.0 ± 1.9 (16.0 - 51.0)	28.9 ± 2.0 (14.0 - 44.0)	31.0 ± 1.9 (11.0 - 44.0)	0.52
**PANAS negative (score /50)**	15.0 [12.0 - 20.0] (10.0 - 33.0)	13.5 [11.0 - 16.5] (9.0 - 26.0)	13.0 [11.0 - 16.5] (10.0 - 24.0)	0.33
**Sleep duration previous night (hours)**	6.8 [6.0 - 7.8] (4.2 - 12.0)	7.5 [7.0 - 7.5] (6.0 - 10.0)	6.5 [5.2 - 7.6] (4.3 - 9.3)	0.16
**Time since supper to fMRI scan (hours)**	16.4 [15.7 - 17.0] (14.8 - 19.1)	16.1 [15.6 - 16.7] (14.9 - 20.3)	16.5 [16.0 - 17.3] (15.0 - 18.6)	0.41
**Absolute motion during food task (mm)**	0.24 [0.19 - 0.38] (0.13 - 1.09)	0.37 [0.25 - 0.50] (0.1 - 0.9)	0.36 [0.26 - 0.52] (0.17 - 1.03)	0.13
**Relative motion during food task (mm/TR)**	0.10 [0.08 - 0.13] (0.05 - 0.22)	0.07 [0.15 - 0.09] (0.05 - 0.23)	0.11 [0.08 - 0.13] (0.06 - 0.36)	0.66
**Absolute motion during Audio-Motor-Visual task (mm)**	0.23 [0.17 - 0.43] (0.09 - 1.25)	0.28 [0.14 - 0.44] (0.09 - 0.91)	0.20 [0.19 - 0.37] (0.09 - 1.20)	0.99
**Relative motion during Audio-Motor-Visual task (mm/TR)**	0.09 [0.07 - 0.12] (0.05 - 0.22)	0.10 [0.07 - 0.12] (0.05 - 0.39)	0.09 [0.08 - 0.12] (0.06 - 0.35)	0.79

Data are presented as mean ± SEM or median [interquartile range] for data that is not normally distributed, and (range).

[a] P value for overall comparison of averages between groups using ANOVA.

Abbreviations: BAND: gastric banding group, BMI-M: body mass index matched group, mm: millimeters, PANAS: positive and negative affect schedule, RYGB: gastric bypass, TR: repetition time. VAS: visual analogue scale.

SUPPLEMENTAL METHODS

Participants

Obese patients who had previously undergone gastric bypass (RYGB) or gastric banding (BAND) surgery were recruited between June 2009 and June 2011 from the Imperial Weight Centre, Charing Cross Hospital, London, UK at follow-up clinics or through invitation letters. A BMI-matched unoperated control group was recruited from the clinic or by public advertisement. The study was approved by the Local Research Ethics Committee, performed in accordance with the principles of the Declaration of Helsinki. All participants provided written informed consent.

Exclusion and inclusion criteria

Inclusion criteria for the study were: for surgical groups (i) loss of more than 8% of their total body weight since surgery, and (ii) surgery more than 2 months ago. All surgical procedures were performed by one of two surgeons (A.A. and T.O.), with RYGB as previously described (Olbers et al. 2003).

Exclusion criteria for the study were: (i) smoking, (ii) pregnancy or breast feeding, (iii) significant neurological, psychiatric or cardiovascular disease including addiction, stroke and epilepsy, other than previous depression, (iv) commencement of anti-depressants less than 6 months ago, (v) type 2 diabetes mellitus (T2DM) treated with agents other than metformin alone, (vi) type 1 diabetes mellitus.

Exclusion criteria for the scanning visit were: (i) inability to use right-handed button keypad, (ii) claustrophobia, (iii) shoulder width >58cm (inability to fit in scanner bore), (iv) metal implants which would preclude safe MRI scanning, (v) vegetarianism or veganism, (vi) reported gluten or lactose intolerance, and (vii) non-Western diet assessed by dietary record.

Patient characteristics

Eligible subjects attended an initial assessment visit during which they completed a medical history, physical examination and questionnaires to assess mood, psychological traits and eating behaviour. Medical notes were examined to ascertain pre-operative clinical information including body weight, presence of T2DM, and binge eating disorder (BED) from review by the clinic psychiatrist (S.S.) or psychologist, and calculation of obesity co-morbidity score using the Kings criteria (Aylwin & Al-Zaman 2008).

In line with standard policy of the obesity clinic, patients in this study had chosen themselves which surgical procedure to undergo. There was therefore no specific selection bias introduced by medical professionals as to which patients had which surgery, as there were no evidence based guidelines to inform bariatric procedure selection. However, in practice patients with T2DM tended to choose RYGB more often due to its more beneficial effects on glycemic control and T2DM resolution (Kashyap et al. 2010, Pournaras et al. 2012). There was therefore a significantly greater prevalence of T2DM and thus obesity co-morbidity score in the RYGB group, but no significant difference in current post-operative T2DM prevalence or other characteristics between surgical groups (see Table 1 and Table S1).

Psychological and eating behaviour questionnaires

The following questionnaires were completed at the initial assessment visit:

1. Dutch Eating Behaviour Questionnaire (DEBQ): to measure dietary restraint, emotional (e.g. stress-induced eating) and external (e.g. food palatability) influences on eating behavior (van Strien 1986).

2. Eating Disorder Examination Questionnaire (EDE-Q): to measure dietary restraint, preoccupation with weight and shape, and binge eating (Fairburn & Beglin 1994).
3. Positive and Negative Affect Schedule (PANAS): to measure symptoms of positive and negative affect over the previous week, which have previously been correlated with fMRI responses to food pictures (Watson et al. 1988, Killgore & Yurgelun-Todd 2006).
4. Beck Depression Inventory (BDI-II): to identify symptoms of depression (Beck et al. 1996)
5. Barratt Impulsivity Scale: to measure impulsivity which has been linked to overeating (Patton et al. 1995, Schag et al. 2013).
6. Eysenck Personality Questionnaire (EPQ-R): to measure extraversion, psychoticism, neuroticism and tendency to lying (Eysenck 1985).
7. Behavioural Activation / Behavioural Inhibition Scales (BAS/BIS): to measure punishment and reward sensitivity. BIS/BAS (reward responsiveness) scores have previously been correlated with fMRI responses to food pictures (Carver & White 1994, Beaver et al. 2006).

Scanning visit protocol

On the day before scanning, subjects were instructed to avoid exercise and alcohol intake, to eat their usual supper at 8.00pm, and then attend the Sir John McMichael Centre Clinical Investigation Unit in the morning having eaten nothing since supper the evening before. Subjects had measurements of height, weight, % body fat by bio-electrical impedance analysis (Bodystat 1500, Isle of Man, UK), and completed the Positive and Negative Affect Schedule (PANAS) to measure mood over the preceding week. Visual analogue scale (VAS) ratings (0-10 cm) of appetite and other symptoms were recorded at serial time points to measure hunger, pleasantness to eat, volume of food wanting to eat, fullness, sickness, sleepiness and stress (Flint et al. 2000, Blundell et al. 2010).

The visit protocol is illustrated in Figure S1. Area under the curve (AUC) for VAS ratings were calculated from +40 to +150 mins to cover the period over the MRI scan in all three groups; and post-prandial changes in VAS ratings were calculated as delta AUC from baseline at +150 to +240 mins in the two surgical groups.

fMRI protocol

Patients were asked to refrain from strenuous exercise and alcohol the day before and day of the study. Patients were scanned for 1 hour starting between 11am and noon (Goldstone et al. 2009). Female participants were scanned in first half phase of menstrual cycle (apart from one BMI-matched control subject who was scanned on day 16 of her cycle) to avoid variations in reward responses including food over the menstrual cycle (Frank et al. 2010). Pregnancy was excluded at each visit.

fMRI confounding variables

There were no significant differences between the three groups in BMI, % body fat, time since last meal, sleep duration the night before the visit (Benedict et al. 2012, St-Onge et al. 2012), or positive or negative affect (PANAS) at the scanning visit (Table S10) (Killgore & Yurgelun-Todd 2006). During scanning there were no significant differences between the groups in absolute or relative head motion during the food evaluation or auditory-motor-visual fMRI tasks (Table S10).

fMRI acquisition

Whole-brain fMRI data were acquired on a 3T Philips Achieva MRI scanner (Robert Steiner MRI Unit, Hammersmith Hospital, London, UK) with T2* weighted gradient-echo echoplanar imaging with an automated higher-order shim procedure: 44 ascending contiguous 3.25 mm thick slices, 2 x 2 mm voxels; SENSE factor 2 repetition time (TR) 3000 ms; echo time (TE) 30 ms; 90^{0} flip angle;

FOV 190x219, matrix 112x112, slice acquisition angle -30^0 from AC-PC line to reduce frontal lobe signal drop out (Deichmann et al. 2003).

High-resolution T1-weighted turbo field echo structural scans were also collected: (TE 4.6 ms; TR 9.7 ms; flip angle 8°; FOV 240 mm; voxel dimensions, 0.94 x 0.94 x 1.2 mm). B_0 field maps were used to correct for geometric distortions caused by inhomogeneities in the magnetic field as follows: TR 29 ms; TE 3.6ms, 30^0 flip angle; FOV 190 x 219, 44 ascending contiguous 3.25mm thick slices, 2 x 2 mm voxels, ∂TE 0 and 2.5.

Food picture evaluation fMRI paradigm

During the fMRI food picture paradigm, four types of colour photographs were presented in a block design split across two 9 minute, 192 volume runs: (1) 60 high-calorie foods (e.g. pizza, cakes and chocolate), (2) 60 low-calorie foods (e.g. salads, vegetables, fish), (3) 60 non-food related household objects (e.g. furniture, clothing) and (4) 180 Gaussian blurred images of the other pictures (as a low-level baseline), similar to those used previously (Goldstone et al. 2009). Food images were selected to represent familiar foods that are typical to the modern Western diet. Pictures were obtained from freely available websites and the International Affective Picture System (IAPS, NIMH Center for the Study of Emotion and Attention, University of Florida, Gainesville, FL, USA). Food and object pictures were of similar luminosity and resolution.

Each run contained different pictures in 5 blocks each of high-calorie and low-calorie foods and objects interleaved with 31 blocks of blurred pictures (6 pictures per 18 secs) using one of four pseudorandom block orders with a randomized picture order within each block. Every image was displayed for 2500 ms, followed by a 500 ms inter-stimulus interval of a fixation cross. Each high-

calorie food block consisted of equal numbers of foods containing chocolate, non-chocolate sweet and savory non-sweet foods (2 of each).

Images were viewed via a mirror mounted above an 8 channel RF head coil which displayed images from a projector using the IFIS image presentation system (In Vivo, Wurzburg, Germany) and ePrime 2 software (Psychology Software Tools Inc., Pittsburgh, PA, USA). Whilst each image was on display to subjects in the scanner, they were asked to immediately and simultaneously rate how 'appealing' each picture was to them using a 5 button hand-held keypad (1=not at all, 2=not really, 3=neutral, 4=a little, 5=a lot) (Goldstone et al. 2009). The appeal rating was thus made and recorded simultaneously with the stimulus presentation used for fMRI activation.

In our fMRI paradigm we studied the differences in BOLD activation to food pictures between surgical groups, rather than food receipt itself. fMRI paradigms with food pictures have been widely used to study human eating behavior (Carnell et al. 2012), and allow exposure to more complex, real-life food stimuli than can be achieved with the restricted nature of tastants such as milkshakes. Furthermore qualitatively similar correlations of fMRI responses to food pictures, anticipation of food receipt and actual food receipt have been reported (Stice et al. 2013). Furthermore our study has demonstrated that greater activation of brain reward systems during evaluation of high-calorie food pictures is associated with greater palatability of high-calorie foods when actually consumed (see Results - Correlation between outcome measures).

Food pictures

The total caloric load, caloric density and macronutrient composition of the food pictures used in the fMRI task were assessed using Dietplan6 (Foresfield Software Ltd, West Sussex, UK) - high-calorie foods: 834 ± 100 kCal, 321 ± 13 kCal/100g, 42 ± 2 % fat, 48 ± 1 % carbohydrate, 10 ± 1 %

protein; low-calorie foods: 157 ± 18 kCal, 64 ± 5 kCal/100g, 35 ± 3 % fat, 35 ± 3 % carbohydrate, 29 ± 3 % protein; high-calorie vs. low-calorie foods: $P<0.001$ for energy content, density, % protein and % carbohydrate; and $P=0.03$ for % fat (unpaired t-test).

Auditory-motor-visual control fMRI paradigm

A 6 min, 114-volume auditory-motor-visual (AMV) control task was performed. Over nine 33 second blocks, subjects performed two of each of the following tasks simultaneously: (i) listening to a story, (ii) tapping their right index finger once every second, or (iii) watching a 4Hz colour (yellow/blue) flashing checkerboard, with each task performed in 6 blocks, and instructions about whether to start or stop the motor task displayed for 3 seconds prior to each block.

fMRI analysis

The first 6 scans were discarded to allow for the BOLD signal to stabilize. The following preprocessing was applied: motion correction using MCFLIRT (Beckmann et al. 2003), fieldmap-based EPI unwarping using PRELUDE+FUGUE (Woolrich et al. 2004, Chang et al. 2012), non-brain removal using BET (Smith 2002), spatial smoothing using a Gaussian kernel of FWHM 6.0mm, grand-mean intensity normalization of the entire 4D dataset by a single multiplicative factor, and high pass temporal filtering (Gaussian-weighted least-squares straight line fitting, with sigma=100.0s).

Time-series statistical analysis was carried out using FILM with local autocorrelation correction including picture onsets, temporal derivative and motion parameters as co-variates. Two subjects (1 gastric bypass, 1 BMI-matched control) were excluded from fMRI analysis as their average relative motion over the food evaluation or control AMV fMRI tasks was greater than 0.5 mm/TR.

Registration to high resolution T1 structural and/or standard space images was carried out using FLIRT. Registration from high resolution structural to standard space was then further refined using FNIRT non-linear registration (Anderson et al. 2007b, Anderson et al. 2007a).

For the food pictures, higher level analysis was carried out using a fixed effect model to combine the two runs, by forcing the random effects variance to zero in FLAME (FMRIB's Local Analysis of Mixed Effects) to determine activation for the following contrasts: food > objects (high-calorie or low-calorie food), high-calorie food only > objects and low-calorie food only > objects (Beckmann et al. 2003, Woolrich et al. 2004).

Similar time-series statistical analysis was performed for the single run AMV paradigm including the onsets of each task (auditory, motor and visual), with temporal derivative and motion parameters as co-variates, to contrast activation during performance of each task with that when the other tasks were being performed.

All higher-level analysis was carried out using FLAME (FMRIB's Local Analysis of Mixed Effects) stage 1 (Beckmann et al. 2003, Woolrich et al. 2004).

fMRI regions of interest

Functional regions of interest (fROIs) for the following areas: bilateral OFC, amygdala, nucleus accumbens, anterior insula and caudate nucleus (Figure S2) were determined from a separate cohort of 24 overweight/obese subjects (Table S2) who underwent an identical protocol after fasting overnight. Higher level whole brain analysis was carried out with mixed effects analysis to identify those voxels which were significantly more activated at the group level, with correction for multiple comparisons made using false discovery rate (FDR) at P<0.05 for the food>objects

contrast (high-calorie or low-calorie food minus objects) (Table S3). Similar functional localizers were made from this separate cohort for the control auditory, motor and visual tasks for bilateral superior posterior temporal gyrus (auditory), left pre-central gyrus (motor), bilateral lingual gyrus (visual) (Figure S3, Table S3).

The functional anatomically constrained ROIs were obtained by masking these group activation maps with the *a priori* anatomical ROI. These were defined by the relevant bilateral ROIs from the cortical and subcortical structural Harvard FSL atlases thresholded at 10% probability. The OFC fROI included regions in the OFC and frontal pole with $y > 22$ and $z < -6$, since analysis of functional activation in this region demonstrated distinct bilateral clusters overlapping the anatomical Harvard atlas regions (Figure S2). The insula mask was subdivided into the anterior insula ($y > 4$) (Chang et al. 2012).

The average (median) magnitude of bilateral BOLD activation within each *a priori* fROI was then extracted for each individual subject separately for any food, high-calorie food and low-calorie food (> object) contrasts using featquery in FSL, to measure the differences in activation between groups for the different picture categories, or different control auditory-motor-visual tasks. Average BOLD activation for each of these contrasts within each ROI was then compared between groups outside FSL, adjusting for age, gender and BMI.

Food palatability

Ad libitum Hagen Daz™ vanilla or pralines and cream flavoured ice cream, was given to subjects in the operated groups in 50ml (43g) portions every 5 minutes and subjects were asked to eat until comfortably full (le Roux et al. 2007). Upon completion, they were asked to rate by VAS how 'pleasant' and 'sweet' the ice cream test meal was to eat. BMI-M control subjects did not have an

ice cream test meal.

Dietary habits

Diet macronutrient composition was assessed using 3-day self-reported dietary records at home in the two surgical groups and analyzed using Dietplan6 (Foresfield Software Ltd., West Sussex, UK).

Metabolic, hormone and bile acid assays

Blood samples for gut hormone analysis were collected into chilled lithium heparin polypropylene tubes, containing 4-(2-Aminoethyl) benzenesulfonyl fluoride hydrochloride (AEBSF) (A8456 Sigma-Aldrich) and aprotinin (Nordic Phama UK) protease inhibitor to give final concentration of 1 mg/mL and 200 kIU/mL whole blood respectively. Blood samples were centrifuged at 4ºC, 4000 rpm for 10 min. Aliquots of separated plasma were immediately mixed with HCl (final concentration of 0.05M) for subsequent assay of acyl ghrelin, and separate unacidified aliquots for assay of other gut hormones (GLP-1 and PYY). All plasma samples were stored at -80°C until assay. Other metabolic and hormonal assays were done on plain serum or fluoride oxalate plasma samples sent immediately to the routine clinical laboratory.

Plasma glucose and serum insulin were measured in the Department of Clinical Biochemistry, Imperial College Healthcare NHS Trust using either an Abbott Architect ci8200 analyzer (Abbott Diagnostics, Maidenhead, UK) or an Axsym analyzer (Abbott Diagnostics, Maidenhead, UK). Intra-assay coefficients of variation of all measurements were 1.0–5.0%. Plasma GLP-1 (GLP-1 $_{1\text{-}36}$ amide, GLP-1 $_{7\text{-}36}$ amide and GLP-1 $_{9\text{-}36}$ amide) and PYY (total PYY $_{1\text{-}36}$ and PYY $_{3\text{-}36}$) were assayed using established in-house radio-immunoassays (Allen et al. 1984, Kreymann et al. 1987). Plasma acyl ghrelin was measured by a two-site sandwich ELISA in a single run (Liu et al. 2008). Intra-assay coefficients of variation (CV) for gut hormones were <10%.

Extraction of bile acids (BA) from plasma was performed as described previously.(Tagliacozzi et al. 2003) BA fractions were analysed using high-performance liquid chromatography (Jasco, Essex, UK) tandem mass spectrometry (Applied Biosystems, Cheshire, UK). The method was linear between 0.1 and 10 µmol/L for all BAs and their conjugates with CV of 1.5-6.8% at the lower limit of quantitation (0.1 µmol/L). The inter-assay CV was 3.6-8.0%.

Area under the curve (AUC) for metabolites and hormones were calculated from +40 to +150 mins, and for bile acids from +70 to +150 mins, to cover the period before and over the MRI scan in all three groups; and in the two surgical groups post-prandial changes in metabolites, hormones and bile acids were calculated as delta AUC from baseline at +150 to +210 mins per kCal ice cream eaten at lunch.

Dumping symptoms

The presence of symptoms of possible 'dumping syndrome' was assessed using change in nausea and sleepiness from before lunch to 1.5 hours after lunch (ΔAUC +150 to +240 mins), and change in physiological markers indicative of dumping syndrome, pulse and blood pressure, from before lunch to one hour after lunch (difference +150 to +210 min) (Ukleja 2005). In addition patients retrospectively completed two validated questionnaires to assess post-prandial symptoms of dumping (e.g. fainting, breathlessness, sleepiness, palpitations, headaches and nausea) in the 3 months following surgery (Sigstad 1970, Arts et al. 2009).

Role of funders

None of the funding sources have played a role in the collection, analysis, and interpretation or reporting of data or in the decision to submit data for publication.

SUPPLEMENTAL REFERENCES

Allen JM, Fitzpatrick ML, Yeats JC, et al. Effects of peptide YY and neuropeptide Y on gastric emptying in man. *Digestion.* 30:255-62, 1984

Anderson J, Jenkinson M, Smith S. Non-linear-registration, aka Spatial normalisation. 2007a.

Anderson J, Jenkinson M, Smith S. Non-linear optimisation. 2007b.

Arts J, Caenepeel P, Bisschops R, et al. Efficacy of the long-acting repeatable formulation of the somatostatin analogue octreotide in postoperative dumping. *Clin Gastroenterol Hepatol.* 7:432-7, 2009

Aylwin S, Al-Zaman Y. Emerging concepts in the medical and surgical treatment of obesity. *Front Horm Res.* 36:229-59, 2008

Beaver JD, Lawrence AD, van Ditzhuijzen J, et al. Individual differences in reward drive predict neural responses to images of food. *J Neurosci.* 26:5160-6, 2006

Beck AT, Steer RA, Ball R, et al. Comparison of Beck Depression Inventories -IA and -II in psychiatric outpatients. *J Pers Assess.* 67:588-97, 1996

Beckmann CF, Jenkinson M, Smith SM. General multilevel linear modeling for group analysis in FMRI. *NeuroImage.* 20:1052-63, 2003

Benedict C, Brooks SJ, O'Daly OG, et al. Acute sleep deprivation enhances the brain's response to hedonic food stimuli: an fMRI study. *J Clin Endocrinol Metab.* 97:E443-7, 2012

Blundell J, de Graaf C, Hulshof T, et al. Appetite control: methodological aspects of the evaluation of foods. *Obes Rev.* 11:251-70, 2010

Carnell S, Gibson C, Benson L, et al. Neuroimaging and obesity: current knowledge and future directions. *Obes Rev.* 13:43-56, 2012

Carver SC, White TL. Behavioral inhibition, behavioral activation, and affective responses to impending reward and punishment: the BIS/BAS scales. *J Pers Soc Psychol.* 67:319-33, 1994

Chang LJ, Yarkoni T, Khaw MW, et al. Decoding the Role of the Insula in Human Cognition: Functional Parcellation and Large-Scale Reverse Inference. *Cereb Cortex.* 2012

Deichmann R, Gottfried JA, Hutton C, et al. Optimized EPI for fMRI studies of the orbitofrontal cortex. *Neuroimage.* 19:430-41, 2003

Eysenck H, Eysenck MW. 1985. In: Personality and Individual Differences: A Natural Science Approach [Internet]. New York: Plenum.

Fairburn CG, Beglin SJ. Assessment of eating disorders: interview or self-report questionnaire? *Int J Eat Disord.* 16:363-70, 1994

Flint A, Raben A, Blundell JE, et al. Reproducibility, power and validity of visual analogue scales in assessment of appetite sensations in single test meal studies. *Int J Obes Relat Metab Disord.* 24:38-48, 2000

Frank TC, Kim GL, Krzemien A, et al. Effect of menstrual cycle phase on corticolimbic brain activation by visual food cues. *Brain Res.* 1363:81-92, 2010

Goldstone AP, de Hernandez CG, Beaver JD, et al. Fasting biases brain reward systems towards high-calorie foods. *Eur J Neurosci.* 30:1625-35, 2009

Kashyap SR, Bhatt DL, Schauer PR. Bariatric surgery vs. advanced practice medical management in the treatment of type 2 diabetes mellitus: rationale and design of the Surgical Therapy And Medications Potentially Eradicate Diabetes Efficiently trial (STAMPEDE). *Diabetes Obes Metab.* 12:452-4, 2010

Killgore WD, Yurgelun-Todd DA. Affect modulates appetite-related brain activity to images of food. *Int J Eat Disord.* 39:357-63, 2006

Kreymann B, Williams G, Ghatei MA, et al. Glucagon-like peptide-1 7-36: a physiological incretin in man. *Lancet.* 2:1300-4, 1987

le Roux CW, Welbourn R, Werling M, et al. Gut hormones as mediators of appetite and weight loss after Roux-en-Y gastric bypass. *Ann Surg.* 246:780-5, 2007

Liu J, Prudom CE, Nass R, et al. Novel ghrelin assays provide evidence for independent regulation of ghrelin acylation and secretion in healthy young men. *J Clin Endocrinol Metab.* 93:1980-7, 2008

Olbers T, Lonroth H, Fagevik-Olsen M, et al. Laparoscopic gastric bypass: development of technique, respiratory function, and long-term outcome. *Obes Surg.* 13:364-70, 2003

Patton JH, Stanford MS, Barratt ES. Factor structure of the Barratt impulsiveness scale. *J Clin Psychol.* 51:768-74, 1995

Pournaras DJ, Glicksman C, Vincent RP, et al. The role of bile after Roux-en-Y gastric bypass in promoting weight loss and improving glycaemic control. *Endocrinology.* 153:3613-9, 2012

Schag K, Schonleber J, Teufel M, et al. Food-related impulsivity in obesity and Binge Eating Disorder - a systematic review. *Obes Rev.* 2013

Sigstad H. A clinical diagnostic index in the diagnosis of the dumping syndrome. Changes in plasma volume and blood sugar after a test meal. *Acta Med Scand.* 188:479-86, 1970

Smith SM. Fast robust automated brain extraction. *Hum Brain Mapp.* 17:143-55, 2002

St-Onge MP, McReynolds A, Trivedi ZB, et al. Sleep restriction leads to increased activation of brain regions sensitive to food stimuli. *Am J Clin Nutr.* 95:818-24, 2012

Stice E, Burger K, Yokum S. Caloric deprivation increases responsivity of attention and reward brain regions to intake, anticipated intake, and images of palatable foods. *Neuroimage.* 67:322-30, 2013

Tagliacozzi D, Mozzi AF, Casetta B, et al. Quantitative analysis of bile acids in human plasma by liquid chromatography-electrospray tandem mass spectrometry: a simple and rapid one-step method. *Clin Chem Lab Med.* 41:1633-41, 2003

Ukleja A. Dumping syndrome: pathophysiology and treatment. *Nutr Clin Pract.* 20:517-25, 2005

van Strien T, Frijters, J. E. R., Bergers, G. P. A., Defares, P. B. The Dutch Eating Behavior Questionnaire (DEBQ) for assessment of restrained, emotional, and external eating behavior. *Int J Eat Disord.* 5:295–315, 1986

Watson D, Clark LA, Tellegen A. Development and validation of brief measures of positive and negative affect: the PANAS scales. *J Pers Soc Psychol.* 54:1063-70, 1988

Woolrich MW, Behrens TE, Beckmann CF, et al. Multilevel linear modelling for FMRI group analysis using Bayesian inference. *NeuroImage.* 21:1732-47, 2004
